# Supplementary material for: Kinetics of Direct Reaction of Vanadate, Chromate, and Permanganate with Graphene Nanoplatelets for Use in Water Purification
Source: Nanomaterials (Basel). 2024 Jan 8;14(2):140. doi: 10.3390/nano14020140 (PMC10819118; doi:10.3390/nano14020140)
Supplement: Supplementary file 1 [file nanomaterials-14-00140-s001.zip › nanomaterials-2792155-Supporting Material S1 VO3-CrO4-MnO4 wit GNP.pdf]

Supplementary Materials

Kinetics of Direct Reaction of Vanadate, Chromate and Permanganate with Graphene Nanoplatelets for Use in Water Purification

Daniel Konradt [1,*], Detlef Schroden [2], Ulrich Hagemann [3], Markus Heidelmann [3], Hans-Peter Rohns [2], Christoph Wagner [2], and Norbert Konradt [2,*]

[1] Ruhr-Universität Bochum, Fakultät für Maschinenbau und Fakultät für Chemie und Biochemie, Universitätsstraße 150, 44801 Bochum; Germany
[2] Department of Waterworks, Stadtwerke Düsseldorf AG, Wiedfeld 50, 40589 Düsseldorf, Germany; dschroden@swd-ag.de (D.S.); hprohns@swd-ag.de (H.-P.R.); cwagner@swd-ag.de (C.W.)
[3] ICAN, NETZ Building, Carl-Benz-Straße 199,47057 Duisburg; ulrich.hagemann@uni-due.de (U.H.); markus.heidelmann@uni-due.de (M.H.)
* Correspondence: daniel.konradt@rub.de (D.K.); nkonradt@swd-ag.de (N.K.)

S1. Reagents for ICP-MS analytics.

Substance	Supplier	Product numbers
Nitric acid 65%, suprapur	Merck	1.00456.1000
SPETEC 169 calibration standard, 2% HNO3, elements Cu, Zn, As, Ba, Co, Cr, Li, Ni, Pb, Se, Sr, Ti, Be, Ag, Cd, U, V	SPETEC	J2-MEB562067
SPETEC 170 calibration standard, 2% HNO3, elements Al, Fe, Mn	SPETEC	J2-MEB562060
SPETEC 171 calibration standard, 2% HNO3, elements Ca, Na, K, Mg, B	SPETEC	J2-MEB562065
SPETEC 172 calibration standard, 2% HNO3, elements Si, P	SPETEC	J2-MEB562069
Certified Reference Material, Hard Drinking Water UK - Metals	LGC	LGC6026
Deionized water, >18.2 MΩ/cm made with Purelab Classic	ELGA LabWater Veolia Water Technologies Deutschland GmbH, Celle, Germany	

S2. Analytical characteristics of ICP-MS analytics.

Calibration parameter [1]	Chromium, total	Vanadium, total	Manganese, total
Quantification mass (amu)	52	51	55
Calibration range (µg/L)	0.5-24.0	0.5-16.0	3-120
Detection limit (µg/L)	0.6	0.4	1.7
Limit of quantitation (µg/L)	1.2	0.9	3.9
Variation coefficient of method (%)	1.7	1.1	1.1
Correlation coefficient	0.9998	0.9998	0.9999
Relative combined uncertainty (%)	4.6	3.3	4.4

[1] Linear calibration evaluation

S3. Analytical characteristics of UV-vis measurements.

Linear calibration evaluation

**Calibration parameters**	**K_2CrO_4, DW [1]**	**K_2CrO_4, 5 mM NH_4Cl, pH 7.5**
Cuvette (cm)	5	5
Wavelength (nm)	375	375
Function	2.389 x + 0.00095	2.124 x + 0.00182
Calibration range (mmol/L)	0.005 - 0.045	0.005 - 0.045
Detection limit (mmol/L)	0.001	0.001
Limit of quantitation (mmol/L)	0.003	0.003
Variation coefficient of method (%)	2.36	3.20
Correlation coefficient	0.9995	0.9990

[1] DW: Drinking water

**Calibration parameters**	**K_2CrO_4, DW**	**K_2CrO_4, 5 mM NH_4Cl, pH 7.5**
Cuvette (cm)	5	5
Wavelength (nm)	375	375
Function	2.389 x + 0.00095	2.124 x + 0.00182
Calibration range (mmol/L)	0.005 - 0.045	0.005 - 0.045
Detection limit (mmol/L)	0.001	0.001
Limit of quantitation (mmol/L)	0.003	0.003
Variation coefficient of method (%)	2.36	3.20
Correlation coefficient	0.9995	0.9990

**Calibration parameters**	**$KMnO_4$, DW**	**$KMnO_4$, 3.1 mM Na_2SO_4, pH 7.5**
Cuvette (cm)	5	5
Wavelength (nm)	525	525
Function	1.157 x + 0.0018	1.207 x + 0.0052
Calibration range (mmol/L)	0.005 - 0.04	0.005 - 0.04
Detection limit (mmol/L)	0.001	0.001
Limit of quantitation (mmol/L)	0.003	0.003
Variation coefficient of method (%)	2.4	3.1
Correlation coefficient	0.9995	0.9991

S4. Dependence of the UV-vis absorption bands on the pH value.

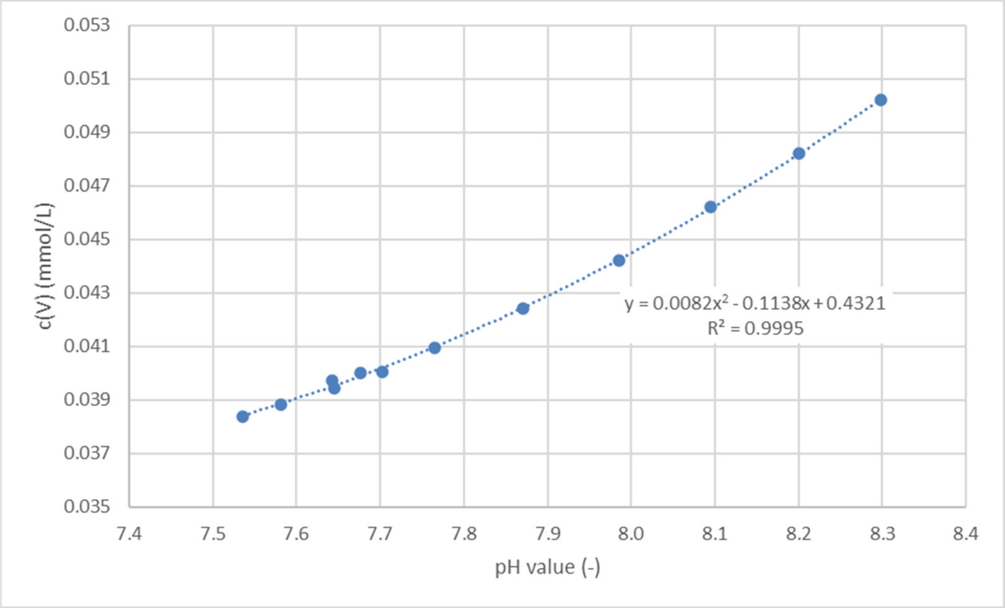

Dependence of the vanadium(V) absorption at 263 nm in DW, expressed as concentration as a function of pH.

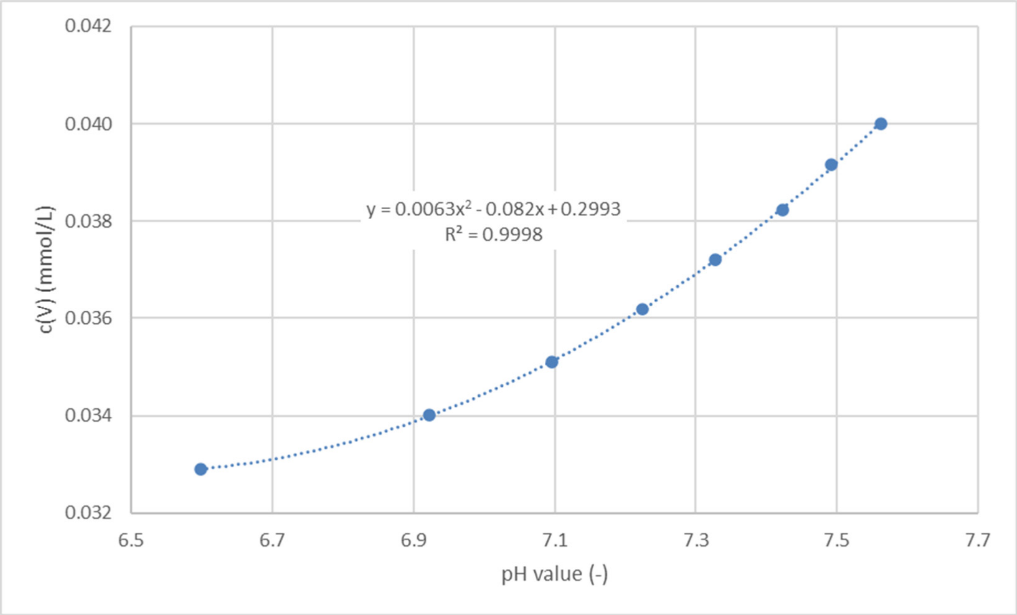

Dependence of the vanadium(V) absorption at 263 nm in 5 mM NH_4Cl, expressed as concentration as a function of pH.

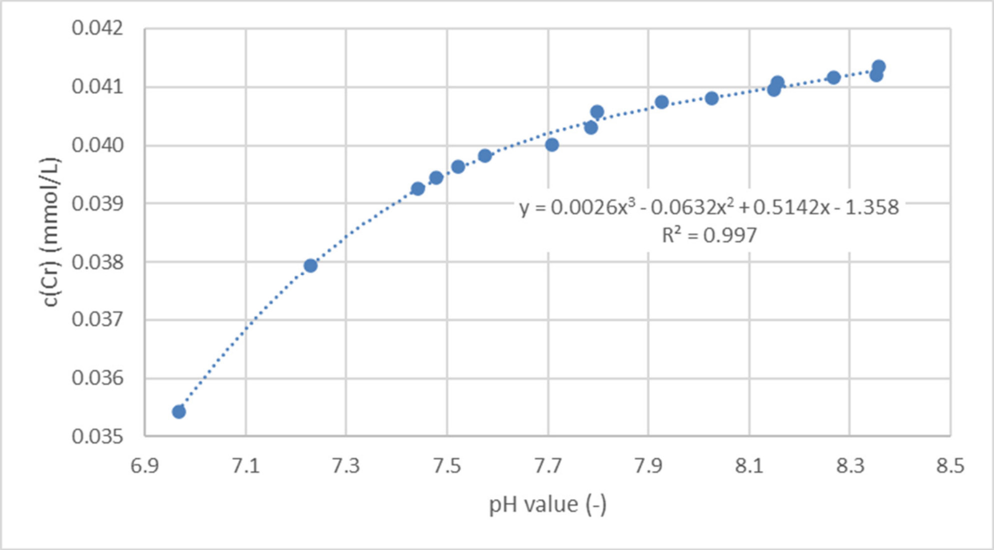

Dependence of the chromium(VI) absorption at 375 nm in DW, expressed as concentration (0.04 mM at pH 7.70) as a function of the pH.

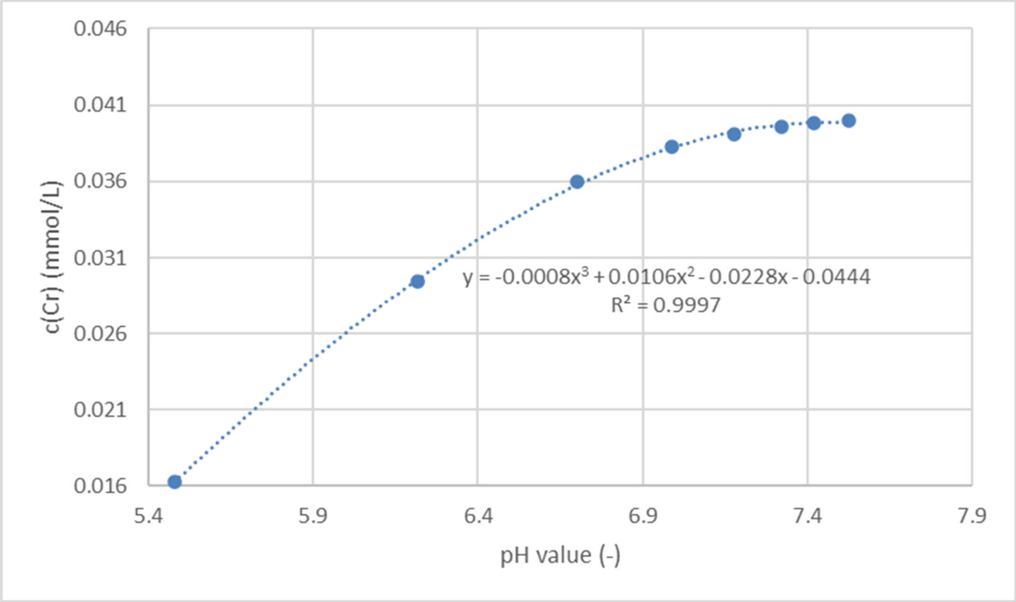

Dependence of the chromium(VI) absorption at 375 nm in 5 mM NH_4Cl, expressed as concentration as a function of pH.

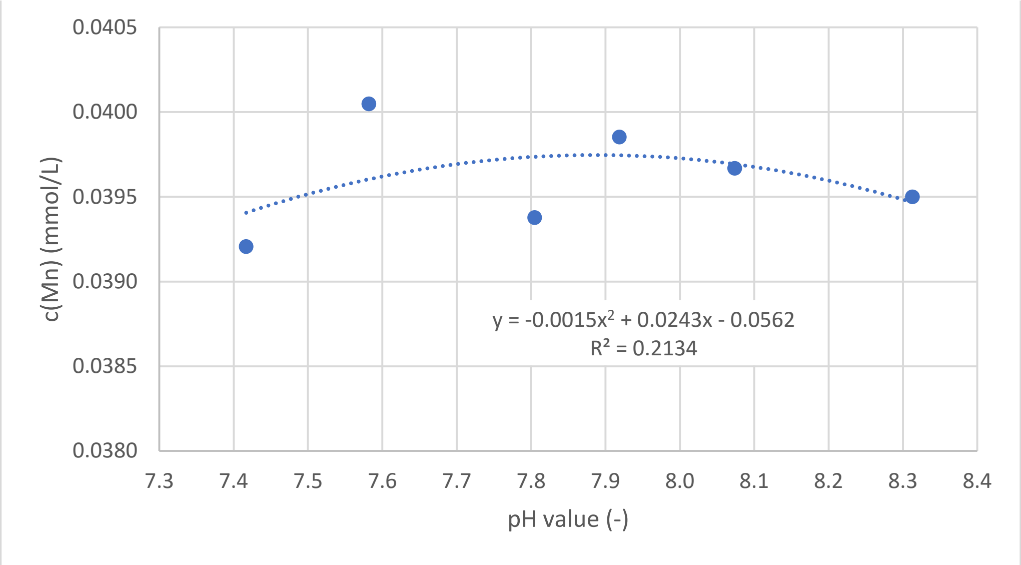

Dependence of the manganese(VII) absorption at 525 nm in DW, expressed as concentration as a function of pH.

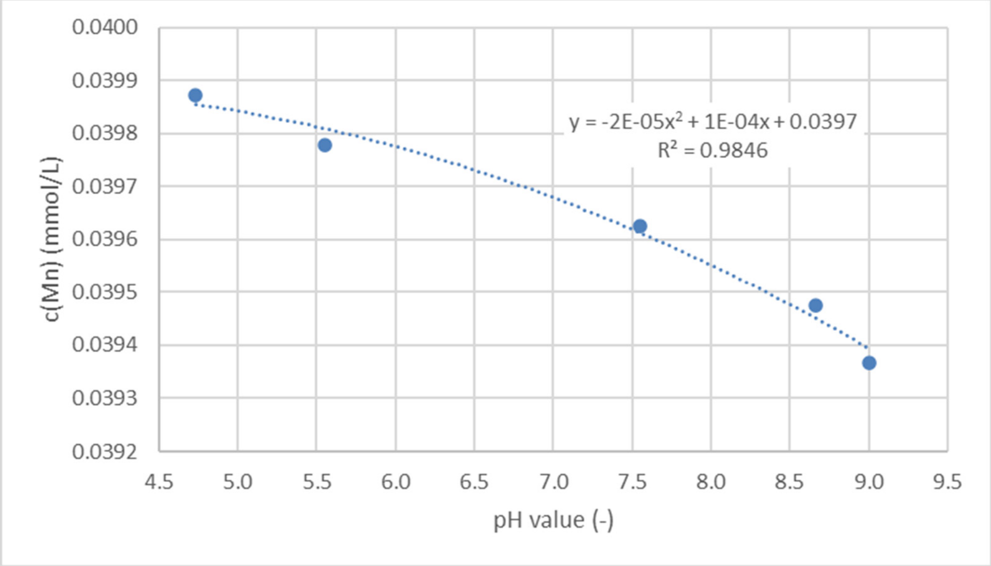

Dependence of the manganese(VII) absorption at 525 nm in 3.1 mM Na_2SO_4, expressed as concentration as a function of pH.

S5. Elemental analysis of GNP and metal-decorated GNP.

Sample	C (%)	H (%)	N (%)	S (%)	K (%)	Mg (%)	Ca (%)	V (%)	Cr (%)	Mn (%)	O2, calc (%)	drying loss (%)
GNP	89.21	0.57	0.89	0.07	0.04	0.03	0.06				9.13	7.71
GNP reg	89.35	0.51	1.04	0.13	0.03	0.03	0.06				8.85	2.24
GNP/$(NH_4)VO_3$ in DW	88.42	0.62	0.88	0.08	0.04	0.06	0.53	0.04			9.33	10.16
GNP reg/$(NH_4)VO_3$ in DW	89.31	0.59	0.94	0.08	0.06	0.03	0.08	0.21			8.70	8.09
GNP/$(NH_4)VO_3$ in NH_4Cl	89.86	0.48	0.97	0.08	0.04	0.03	0.08	0.07			8.39	10.29
GNP/K_2CrO_4 in DW	89.49	0.59	0.87	0.06	0.08	0.03	0.09		0.11		8.68	8.38
GNP reg/K_2CrO_4 in DW	89.72	0.40	0.83	0.07	0.10	0.03	0.08		0.23		8.54	6.98
GNP/K_2CrO_4 in NH_4Cl	89.61	0.50	0.95	0.03	0.07	0.04	0.10		0.11		8.59	39.08
GNP/$KMnO_4$ in Na_2SO_4	89.23	0.46	0.87	0.03	0.08	0.04	0.09			1.02	8.18	45.00

reg: Thermally regenerated; DW: Drinking water

S6. XPS of GNP.

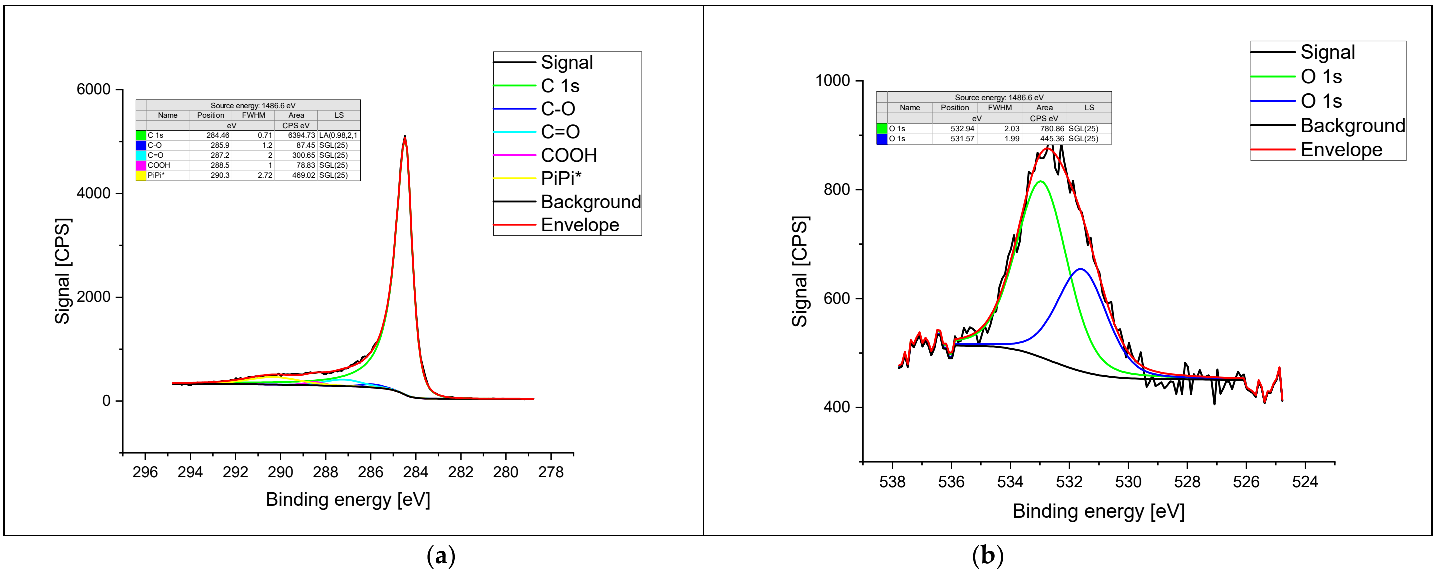

Figure S6. XP spectra of GNP. (a) Carbon (C 1s); (b) Oxygen (O 1s).

S7. TEM of GNP.

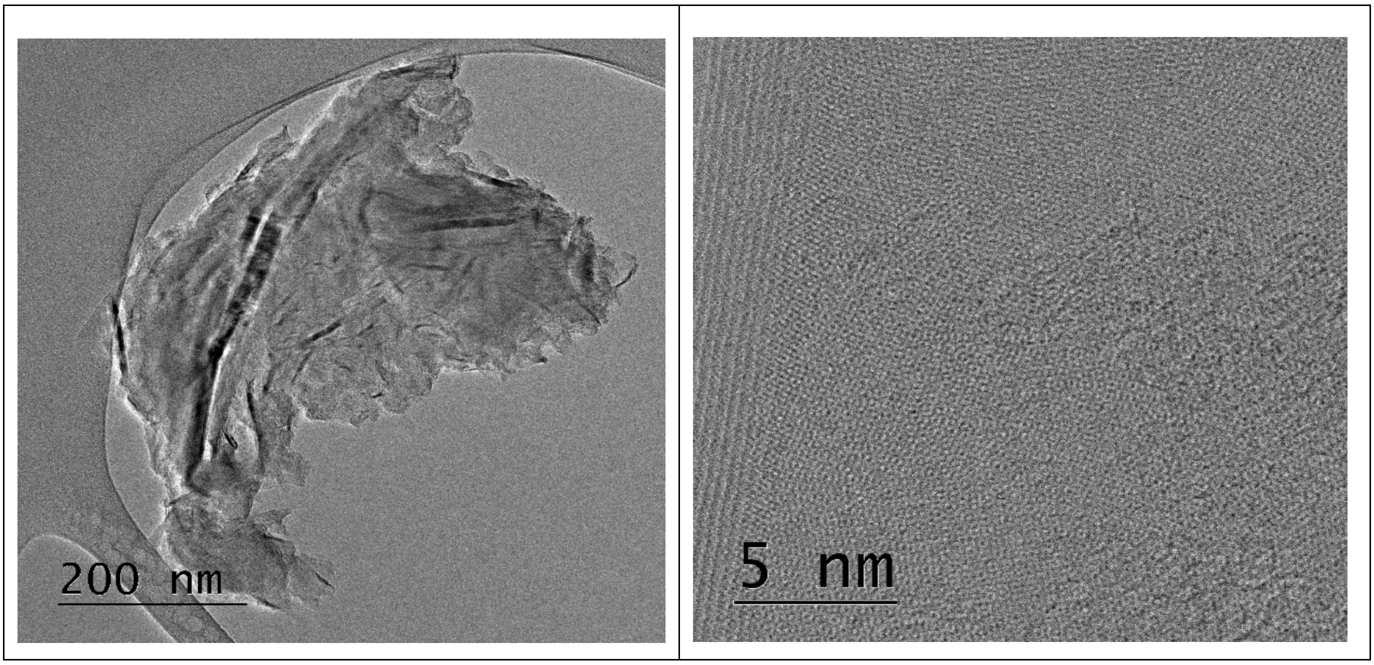

(**a**) (**b**)

Figure S7. TEM of GNP. (a) Single flake; (b) Flake with 11 graphene layers and local disturbed molecular structures.

S8. Raman spectrum of GNP.

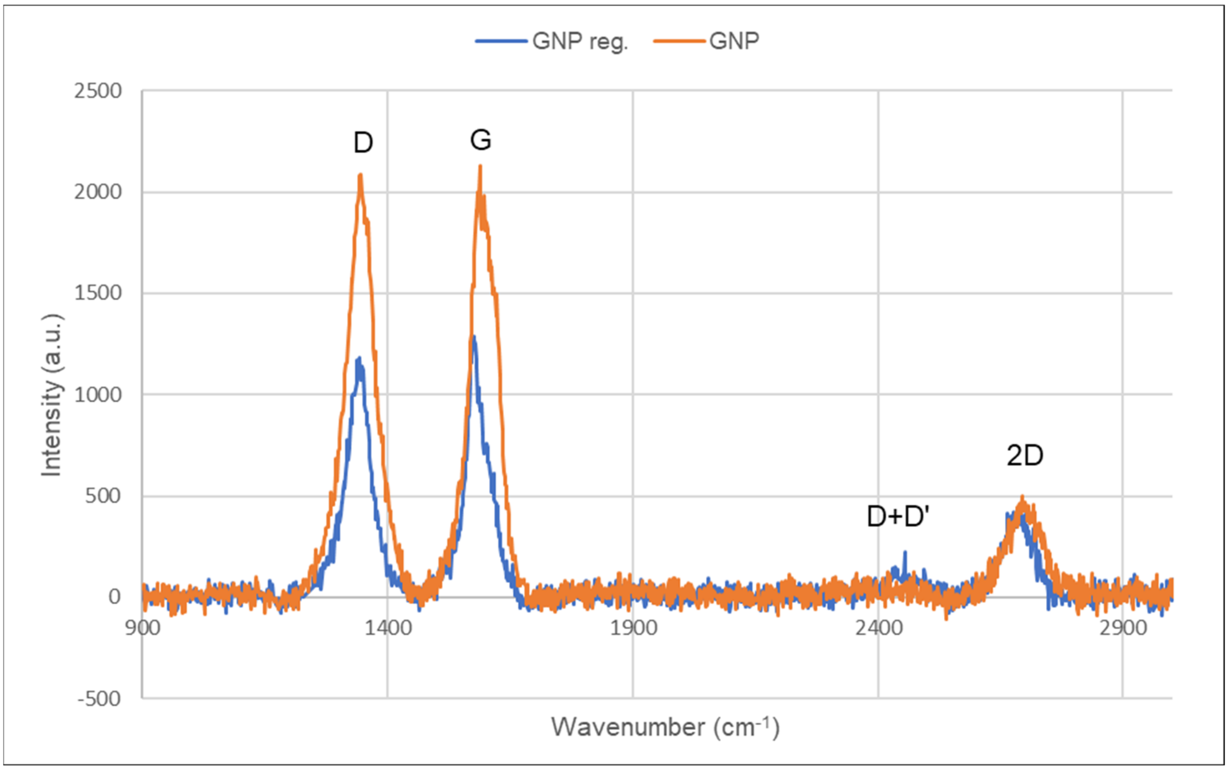

Figure S8. Raman of GNP and regenerated GNP.

S9. IR spectra of GNP.

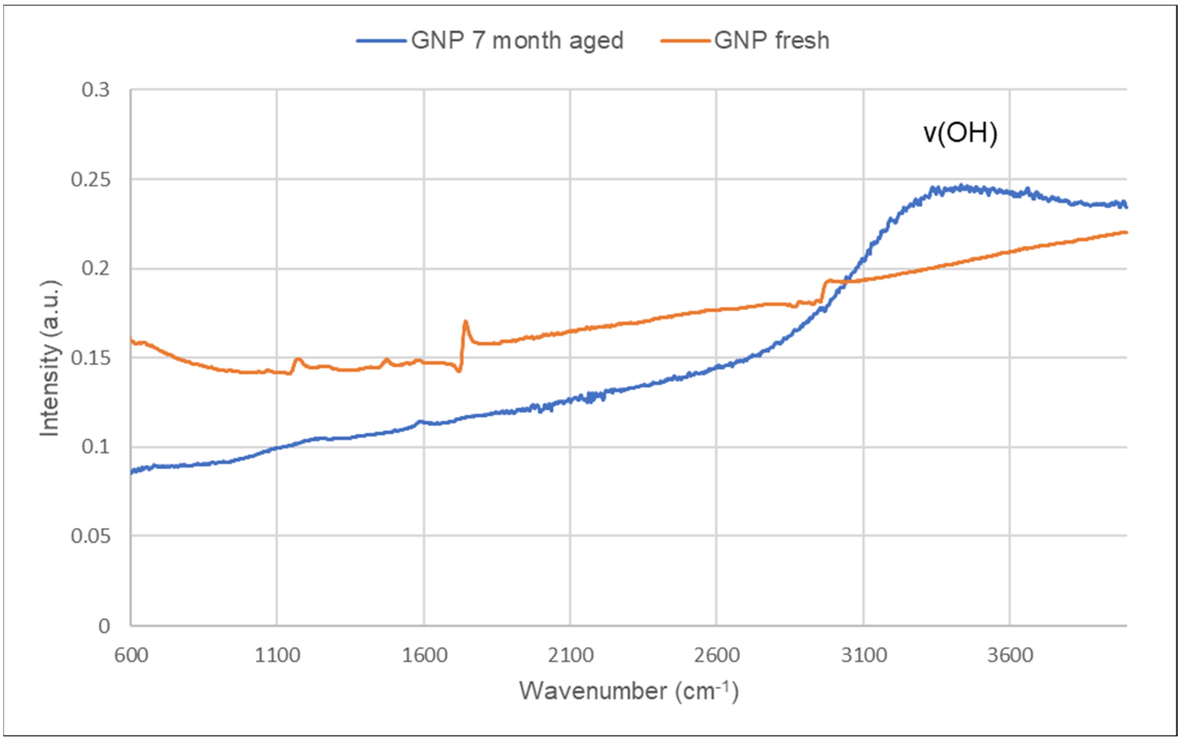

Figure S9. IR spectra of fresh GNP and after 7 month storage.

S10. Weight increase of the GNP upon contact with the ambient air.

Time(h)	Weight increase GNP (%)
0	0.00
1	0.34
2	0.63
3	0.94
4	1.18
5	1.30
6	1.47
24	2.67

S11. Sorption kinetics of oxygen on GNP in water.

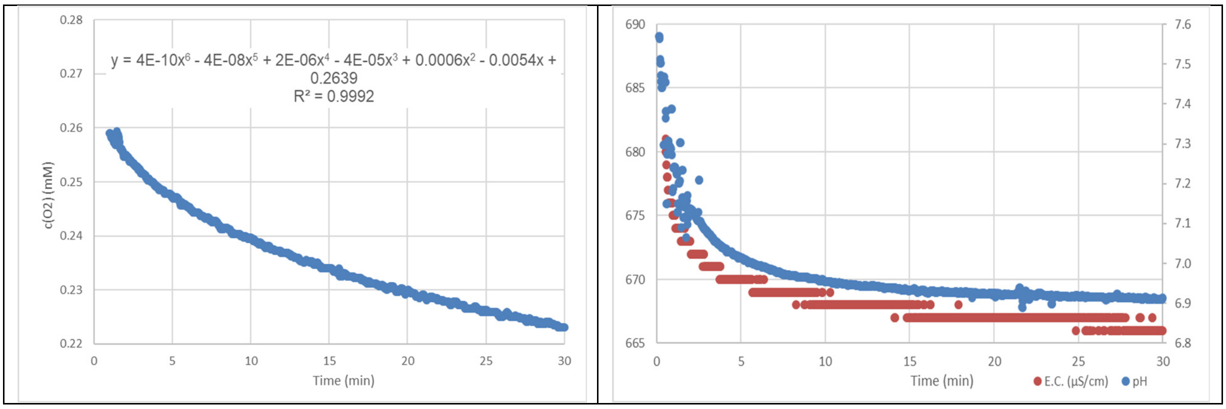

(**a**) (**b**)

Figure S11.1. 0.25 mM O_2 in 150 ml DW with 300 mg GNP. (a) Concentration of O_2 as a function of time with empirical function; (b)E.C. and pH as a function of time, E.C. (left axis), pH (right axis).

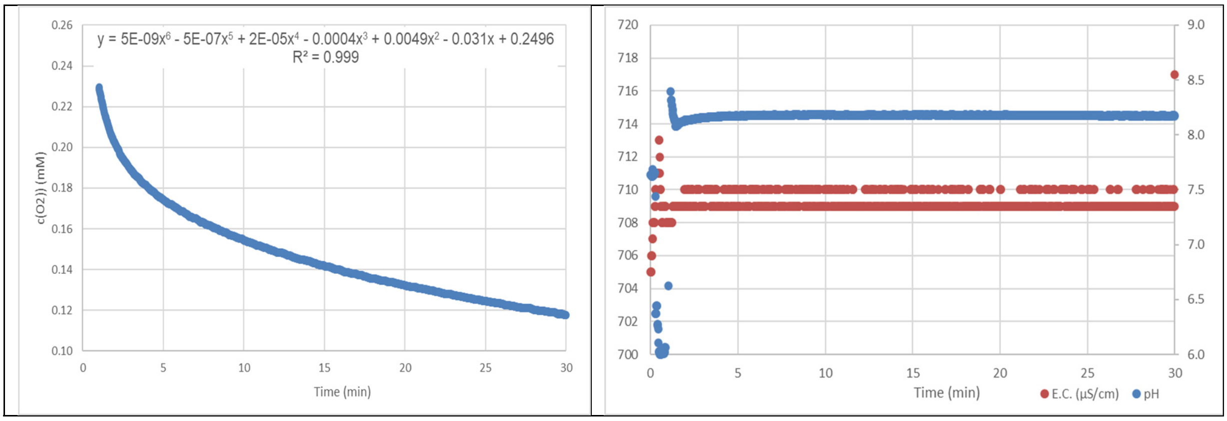

(**a**) (**b**)

Figure S11.2. 0.25 mM O_2 in 150 ml DW with 300 mg GNP reg. (a) Concentration of O_2 as a function of time with empirical function; (b)E.C. and pH as a function of time, E.C. (left axis), pH (right axis).

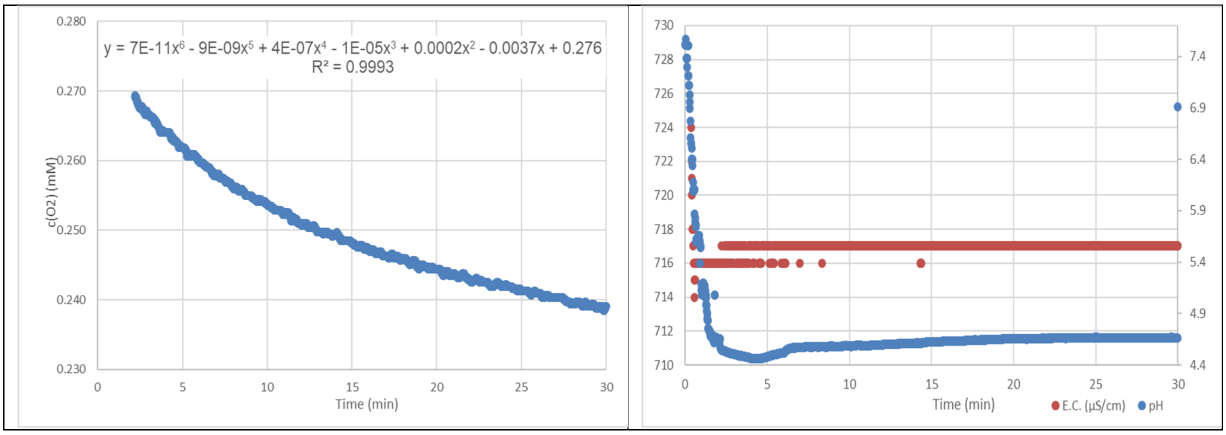

(**a**) (**b**)

Figure S11.3. 0.25 mM O_2 in 150 ml SW with 300 mg GNP. (a) Concentration of O_2 as a function of time with empirical function; (b)E.C. and pH as a function of time, E.C. (left axis), pH (right axis).

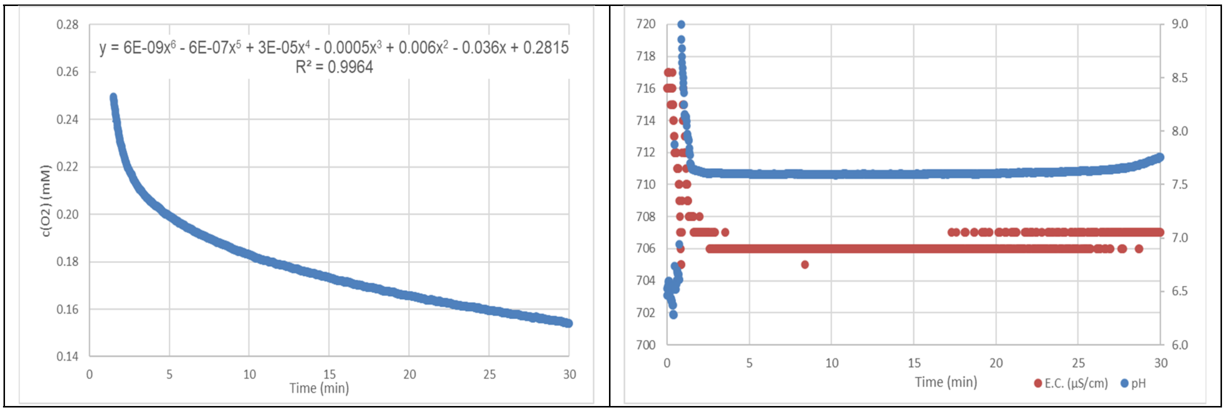

(**a**) (**b**)

Figure S11.4. 0.25 mM O_2 in 150 ml SW with 300 mg GNP reg. (a) Concentration of O_2 as a function of time with empirical function; (b)E.C. and pH as a function of time, E.C. (left axis), pH (right axis).

S12. Sorption kinetics of vanadium(V), chromium(VI) and manganese(VII) on GNP.

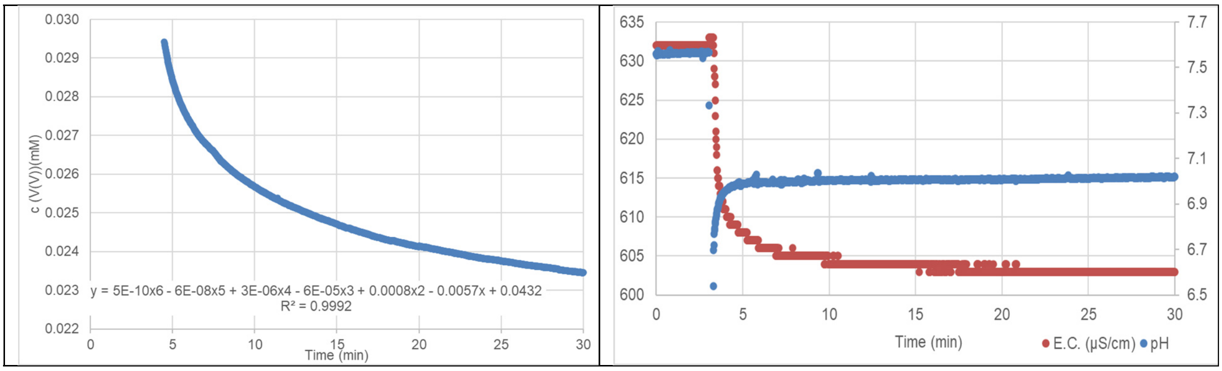

(a) (b)

Figure S12.1 0.04 mM NH_4VO_3 in DW, 500 mg GNP. (a) Concentration of V(V) as a function of time; (b)E.C. and pH as a function of time, E.C. (left axis), pH (right axis).

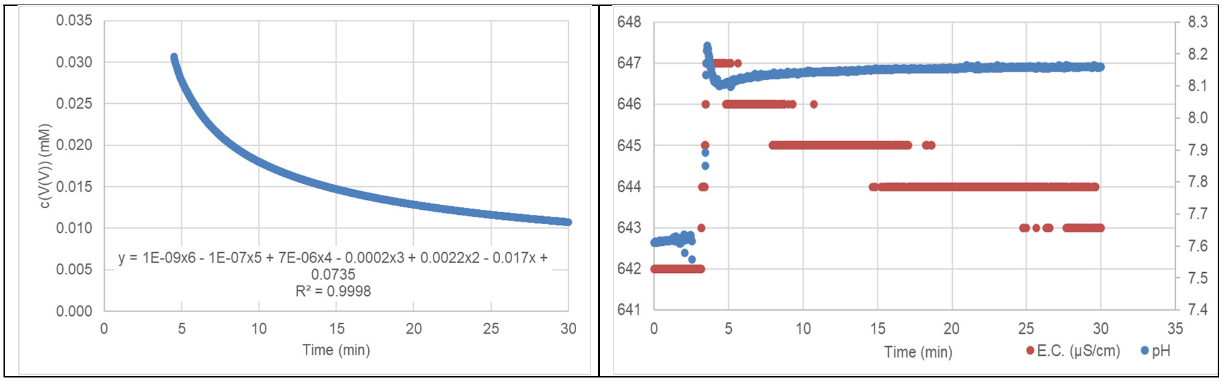

(a) (b)

Figure S12.2. 0.04 mM NH_4VO_3 in DW, 500 mg GNP reg. (a) Concentration of V(V) as a function of time; (b)E.C. and pH as a function of time, E.C. (left axis), pH (right axis).

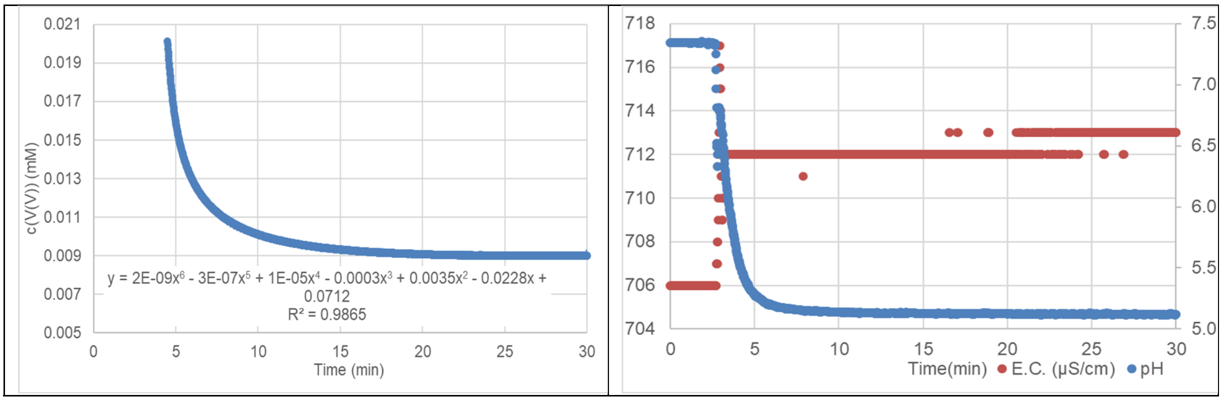

(a) (b)

Figure S12.3. 0.04 mM NH_4VO_3 in 5 mM NH_4Cl, 500 mg GNP. (a) Concentration of V(V) as a function of time; (b)E.C. and pH as a function of time, E.C. (left axis), pH (right axis).

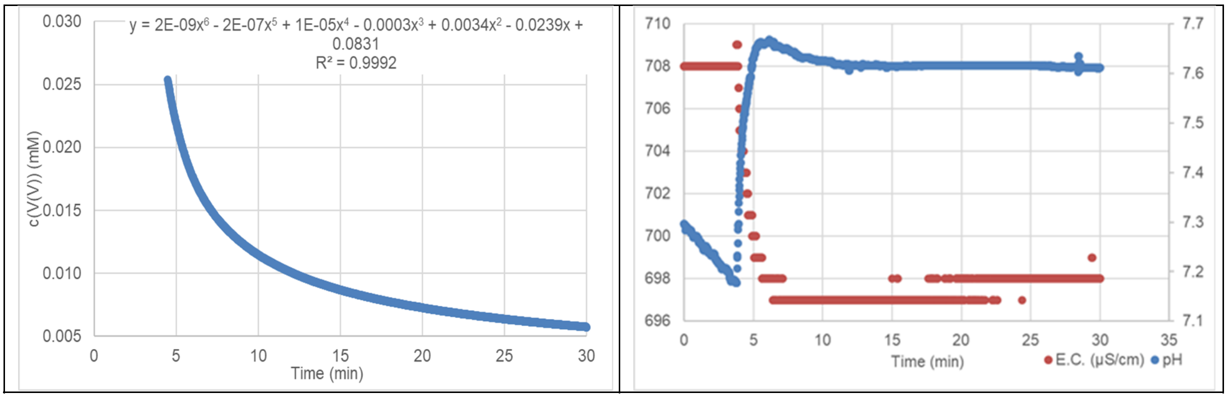

(**a**) (**b**)

Figure S12.4. 0.04 mM NH_4VO_3 in 5 mM NH_4Cl, 500 mg GNP reg. (a) Concentration of V(V) as a function of time; (b)E.C. and pH as a function of time, E.C. (left axis), pH (right axis).

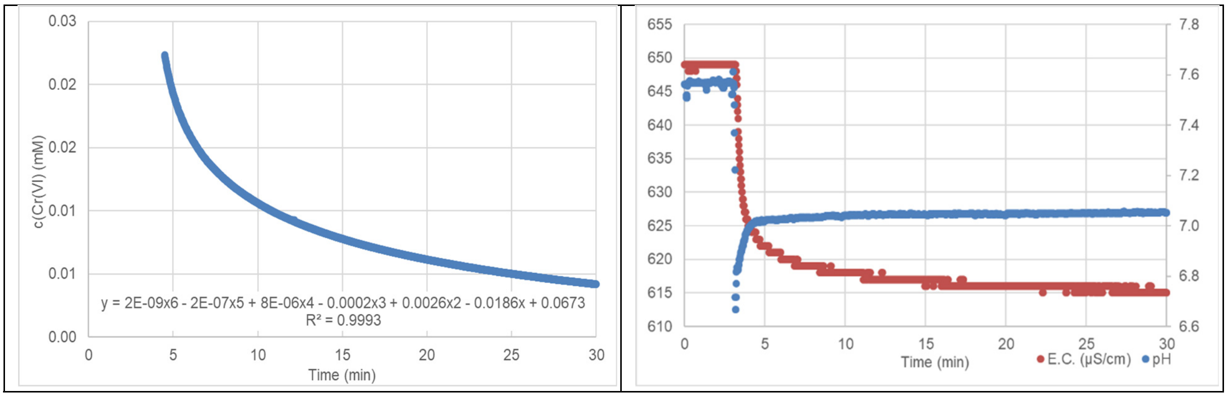

(**a**) (**b**)

Figure S12.5. 0.04 mM K_2CrO_4 in DW, 500 mg GNP. (a) Concentration of Cr(VI) as a function of time; (b)E.C. and pH as a function of time, E.C. (left axis), pH (right axis).

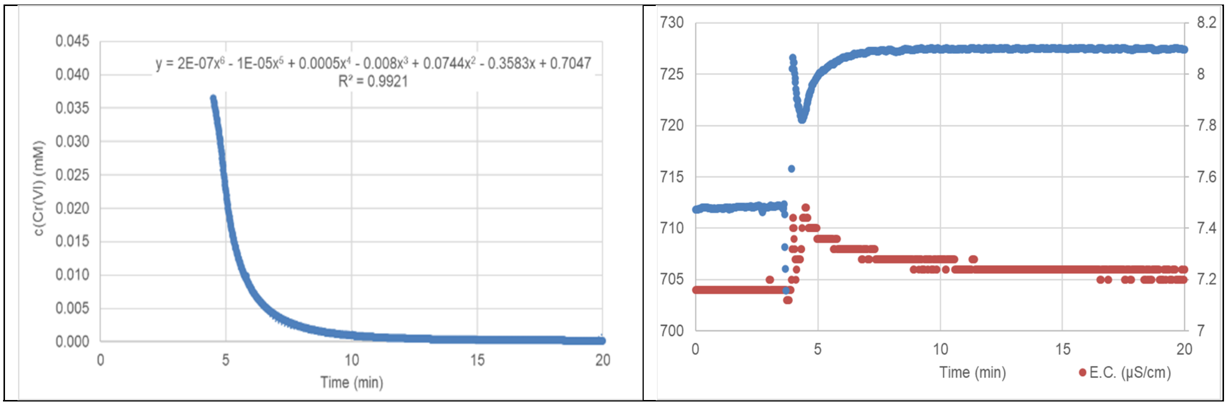

(**a**) (**b**)

Figure S12.6. 0.04 mM K_2CrO_4 in DW, 500 mg GNP reg. (a) Concentration of Cr(VI) as a function of time; (b)E.C. and pH as a function of time, E.C. (left axis), pH (right axis).

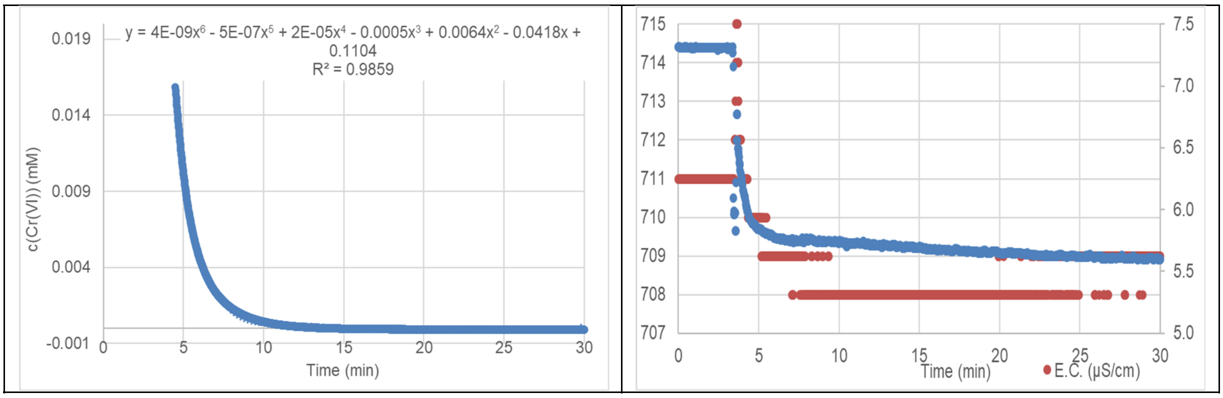

(**a**) (**b**)

Figure S12.7. 0.04 mM K_2CrO_4 in 5 mM NH_4Cl, 507 mg GNP. (a) Concentration of Cr(VI) as a function of time; (b)E.C. and pH as a function of time, E.C. (left axis), pH (right axis).

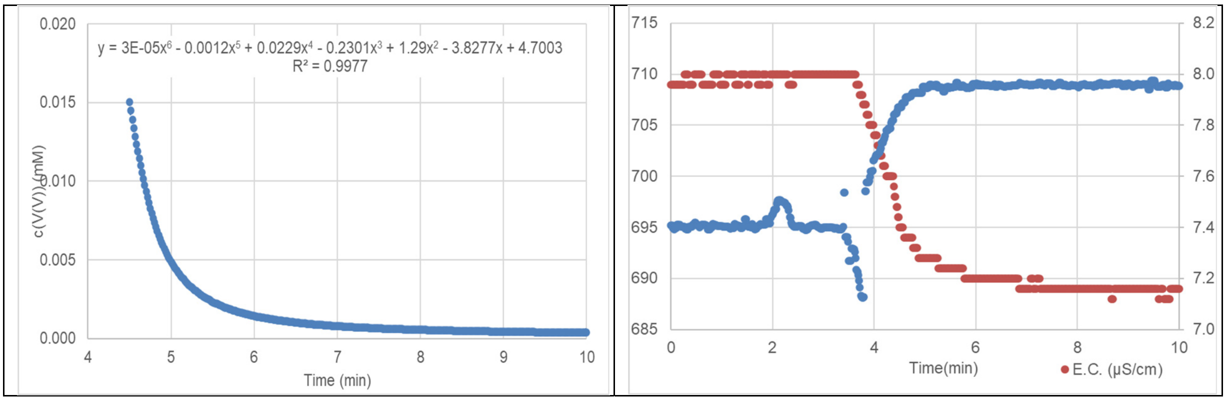

(**a**) (**b**)

Figure S12.8. 0.04 mM K_2CrO_4 in 5 mM NH_4Cl, 500 mg GNP reg. (a) Concentration of Cr(VI) as a function of time; (b)E.C. and pH as a function of time, E.C. (left axis), pH (right axis).

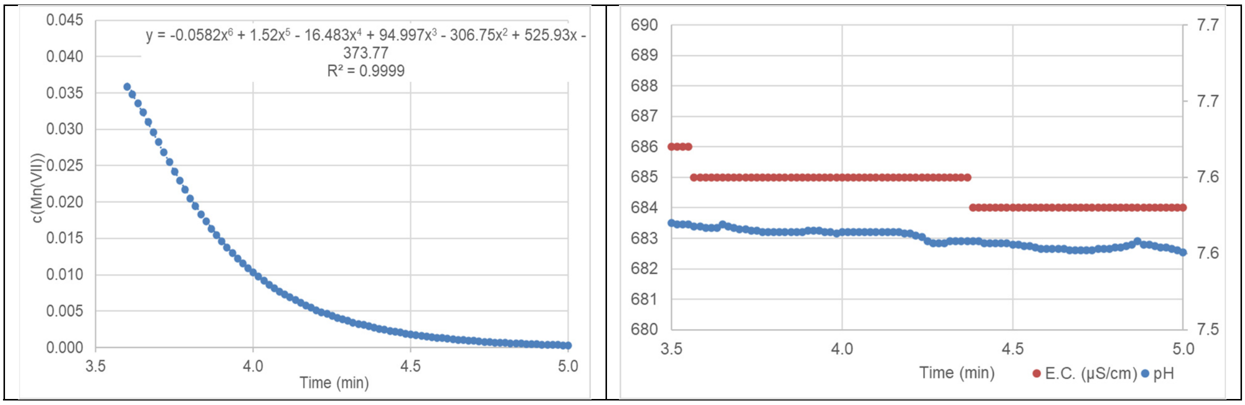

(**a**) (**b**)

Figure S12.9. 0.04 mM $KMnO_4$ in DW, 50.0 mg GNP. (a) Concentration of M(VII) as a function of time; (b)E.C. and pH as a function of time, E.C. (left axis), pH (right axis).

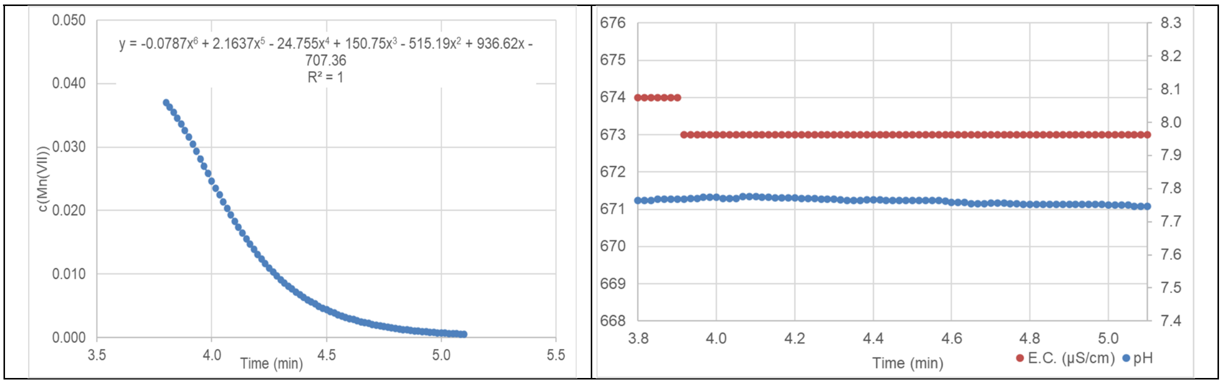

(a) (b)

Figure S12.10. 0.04 mM $KMnO_4$ in DW, 50.0 mg GNP reg. (a) Concentration of Mn(VII) as a function of time; (b)E.C. and pH as a function of time, E.C. (left axis), pH (right axis).

250 ml 3.1 mM Na_2SO_4, 0.04 mM KMnO4, 50.7 mg GNP.

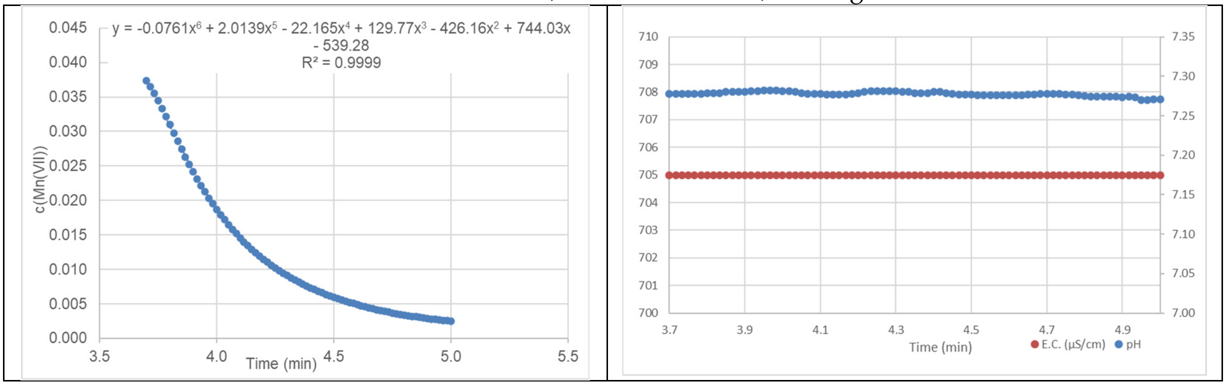

(a) (b)

Figure S12.11. 0.04 mM $KMnO_4$ in 3.1 mM Na_2SO_4, 50.7 mg GNP. (a) Concentration of Mn(VII) as a function of time; (b)E.C. and pH as a function of time, E.C. (left axis), pH (right axis).

250 ml 3.1 mM Na_2SO_4, 0.04 mM $KMnO_4$, 50.9 mg GNP reg.

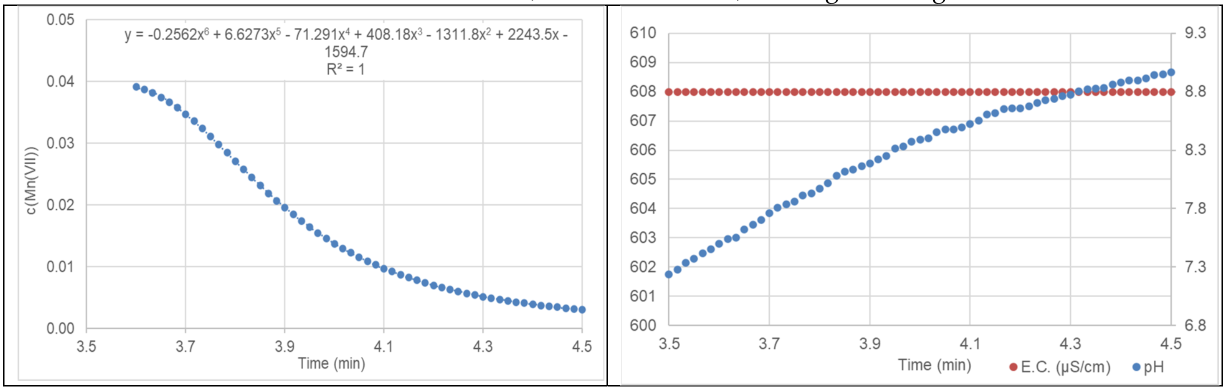

(a) (b)

Figure S12.12. 0.04 mM $KMnO_4$ in 3.1 mM Na_2SO_4, 50.9 mg GNP reg. (a) Concentration of Mn(VII) as a function of time; (b)E.C. and pH as a function of time, E.C. (left axis), pH (right axis).

S13. Residual metal concentrations in water after sorption.

Process	Concentration after 30 min (mM), UV-vis	Concentration (mM), ICP-MS
0.04 mM NH_4VO_3 in 5 mM NH_4Cl, 500 mg GNP reg	0.0057	0.0057
0.04 mM K_2CrO_4 in DW, 500 mg GNP reg	<0.003	0.0008
0.04 mM K_2CrO_4 in 5 mM NH_4Cl, 500 mg GNP reg	0.0033	0.0037
0.04 mM K2CrO4 in DW, 500 mg GNP	0.0010	0.0009
0.04 mM $KMnO_4$ in DW, 50.0 mg GNP	<0.003	0.0040
0.04 mM $KMnO_4$ in DW, 50.0 mg GNP reg	<0.003	0.0020

S14. Process conditions of evaluated kinetic data.

Process	Evaluation time (min)	T_m (°C)	pH start	ΔpH [1]	Δ E.C. (μS/cm) [1]
0.04 mM NH_4VO_3 in DW, 500 mg GNP	5.8 - 16.5	25.4 ± 0.5	7.00	0.01	-4
0.04 mM NH_4VO_3 in DW, 500 mg GNP reg.	8.5 - 17.5	24.1 ± 0.1	8.14	0.02	-2
0.04 mM NH_4VO_3 in 5 mM NH_4Cl, 500 mg GNP	9.1 - 12.8	25.0 ± 0.1	5.14	-0.01	0
0.04 mM NH_4VO_3 in 5 mM NH_4Cl, 500 mg GNP reg	7.4 - 11.5	25.2 ± 0.1	7.65	-0.03	0
0.04 mM K_2CrO_4 in DW, 500 mg GNP	8.0 - 23.0	24.8 ± 0.1	7.03	0.01	-3
0.04 mM K_2CrO_4 in DW, 500 mg GNP reg.	9.4 - 18.0	23.9 ± 0.1	8.10	0.00	-1
0.04 mM K_2CrO_4 in 5 mM NH_4Cl, 507 mg GNP	4.9 - 5.8	25.2 ± 0.1	5.85	-0.05	-1
0.04 mM K_2CrO_4 in 5 mM NH_4Cl, 500 mg GNP reg	5.1 - 5.7	24.3 ± 0.1	7.96	0.01	-1
0.04 mM $KMnO_4$ in DW, 50.0 mg GNP	5.1 - 5.7	24.1 ± 0.1	7.56	-0.01	-1
0.04 mM $KMnO_4$ in DW, 50.0 mg GNP reg.	4.1 - 5.0	24.0 ± 0.1	7.78	-0.02	0
0.04 mM $KMnO_4$ in 3.1 mM Na_2SO_4, 50.7 mg GNP	3.8 - 4.8	25.0 ± 0.1	7.28	0.00	0
0.04 mM $KMnO_4$ in 3.1 mM Na_2SO_4, 50.9 mg GNP reg	3.7 - 4.4	24.1 ± 0.2	7.76	1.12	0

[1] Difference between end and start values.

S15. Error estimation due to pH value variation in kinetic evaluations.

Process	pH start	ΔpH	Δc (mM)	Error (%)	pH start	ΔpH	Δc (mM)	Error (%)
	1st order evaluation				PSO / 2nd order kinetic evaluation			
0.04 mM NH_4VO_3 in DW, 500 mg GNP					7.00	<0.01	<0.00001	<0.001
0.04 mM NH_4VO_3 in DW, 500 mg GNP reg					8.14	<0.01	<0.00001	<0.001
0.04 mM NH_4VO_3 in 5 mM NH_4Cl, 500 mg GNP					5.14	-0.01	0.0002	0.003
0.04 mM NH_4VO_3 in 5 mM NH_4Cl, 500 mg GNP reg					7.65	-0.03	0.00	0.007
0.04 mM K_2CrO_4 in DW, 500 mg GNP					7.03	0.01	0.0001	0.002
0.04 mM K_2CrO_4 in DW, 500 mg GNP reg					8.10	<0.01	<0.00001	<0.001
0.04 mM K_2CrO_4 in 5 mM NH_4Cl, 507 mg GNP					5.85	<0.01	<0.00001	<0.001
0.04 mM K_2CrO_4 in 5 mM NH_4Cl, 500 mg GNP reg					7.96	<0.01	<0.00001	<0.001
0.04 mM $KMnO_4$ in DW, 50.0 mg GNP	7.56	-0.01	<0.00001	<0.001				
0.04 mM $KMnO_4$ in DW, 50.0 mg GNP reg	7.78	-0.02	<0.00001	<0.001				
0.04 mM $KMnO_4$ in 3.1 mM Na_2SO_4, 50.7 mg GNP	7.28	<0.01	<0.00001	<0.001				
0.04 mM $KMnO_4$ in 3.1 mM Na_2SO_4, 50.9 mg GNP reg	7.76	1.21	<0.00001	<0.001				

Note: Error (%) = 100 * (c_{end} - c_{start}) / 0.5 (c_{end} + c_{start})

S16. Sorption kinetics of vanadium(V), chromium(VI) and manganese(VII) in DW with mass variation of GNP.

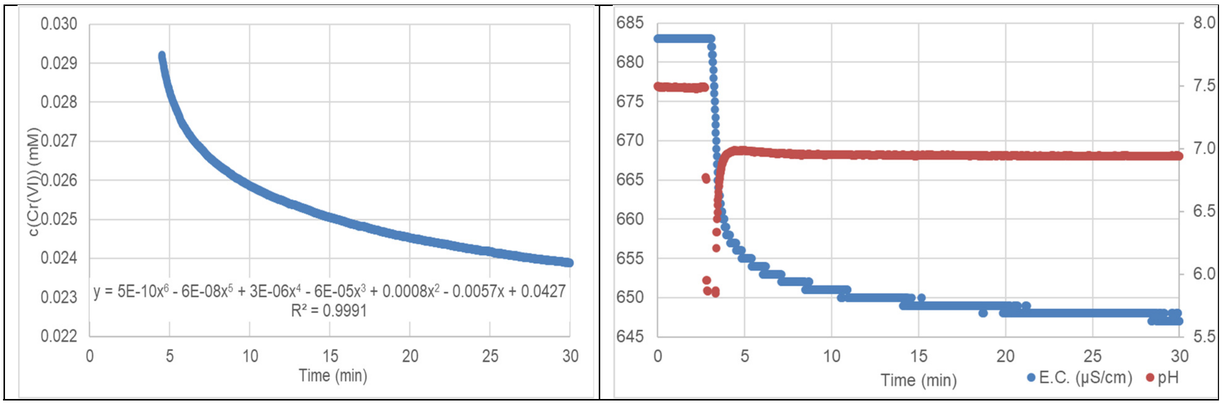

(a) (b)

Figure S16.1 250 ml DW with 0.04 mM NH_4VO_3, 500 mg GNP. (a) Concentration of V(V) as a function of time; (b)E.C. and pH as a function of time, E.C. (left axis), pH (right axis).

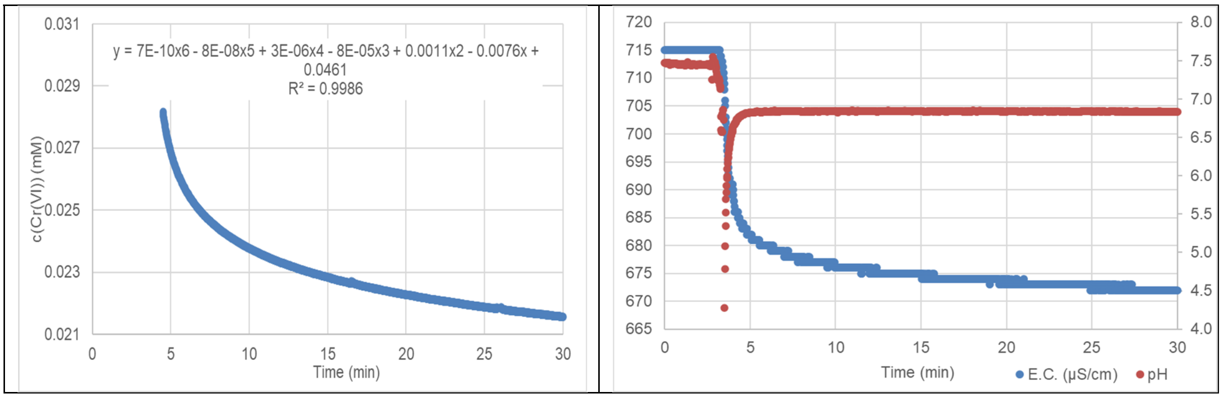

(a) (b)

Figure S16.2 250 ml DW with 0.04 mM NH_4VO_3, 625 mg GNP. (a) Concentration of V(V) as a function of time; (b)E.C. and pH as a function of time, E.C. (left axis), pH (right axis).

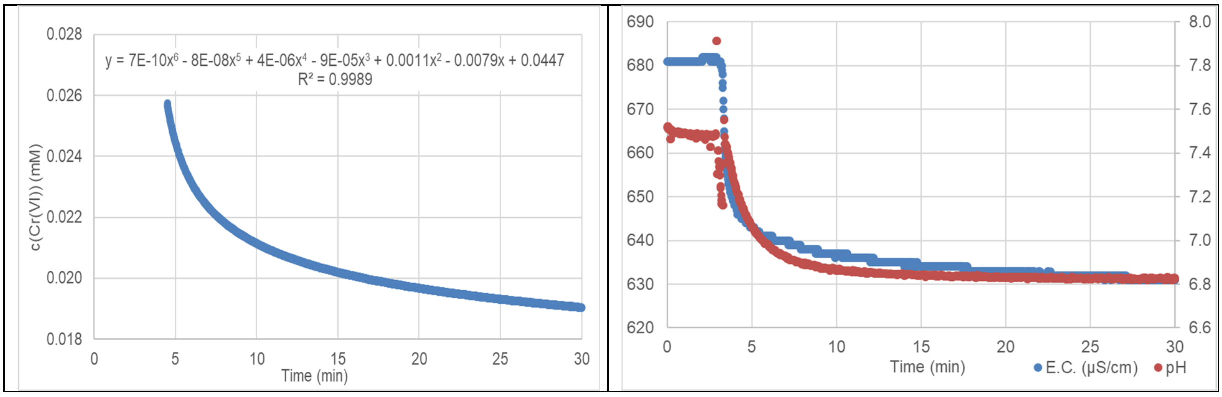

(a) (b)

Figure S16.3 250 ml DW with 0.04 mM NH_4VO_3, 750 mg GNP. (a) Concentration of V(V) as a function of time; (b)E.C. and pH as a function of time, E.C. (left axis), pH (right axis).

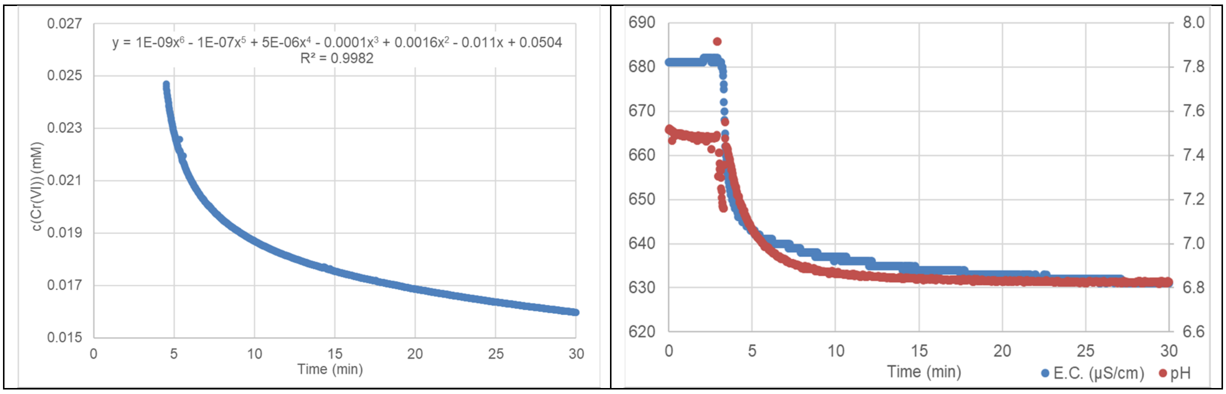

(**a**) (**b**)

Figure S16.4 250 ml DW with 0.04 mM NH_4VO_3, 875 mg GNP. (a) Concentration of V(V) as a function of time; (b)E.C. and pH as a function of time, E.C. (left axis), pH (right axis).

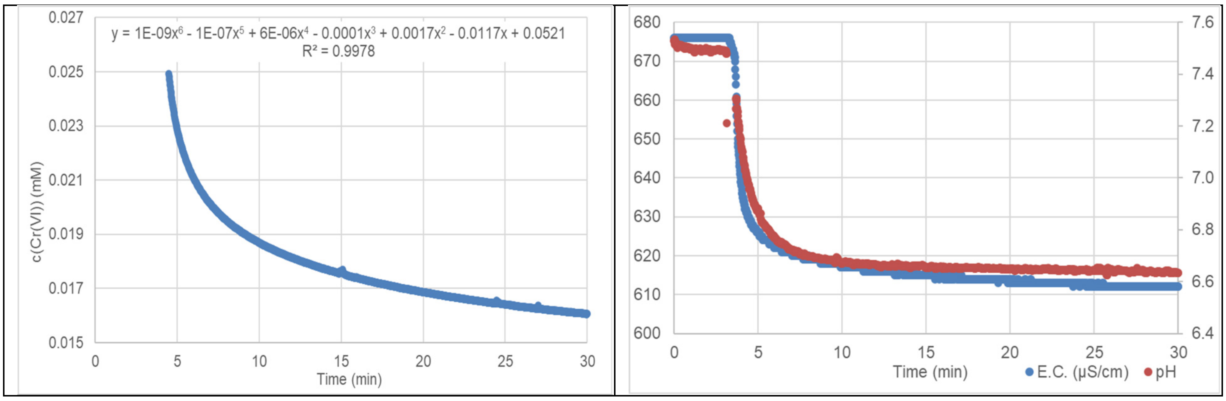

(**a**) (**b**)

Figure S16.5 250 ml DW with 0.04 mM NH_4VO_3, 1000 mg GNP. (a) Concentration of V(V) as a function of time; (b)E.C. and pH as a function of time, E.C. (left axis), pH (right axis).

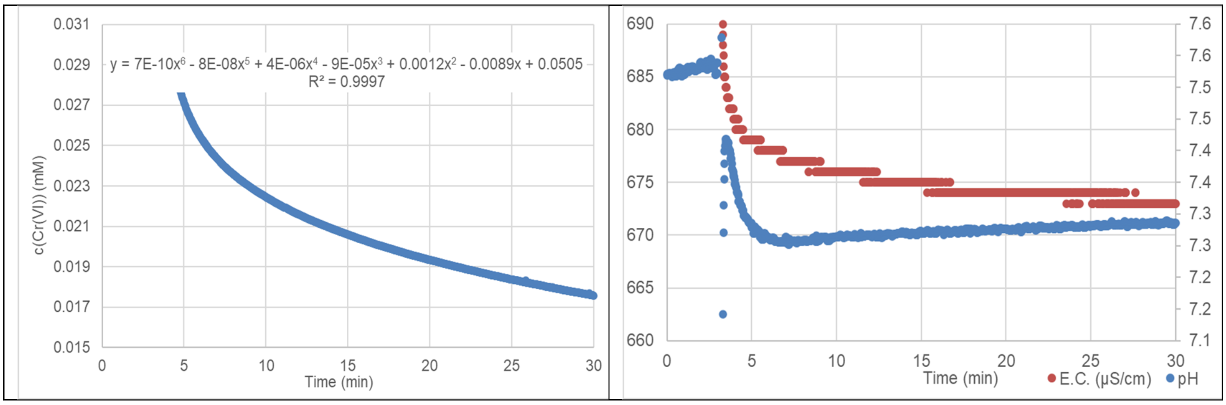

(**a**) (**b**)

Figure S16.6. 250 ml DW with 0.04 mM K_2CrO_4, 250 mg GNP. (a) Concentration of Cr(VI) as a function of time; (b)E.C. and pH as a function of time, E.C. (left axis), pH (right axis).

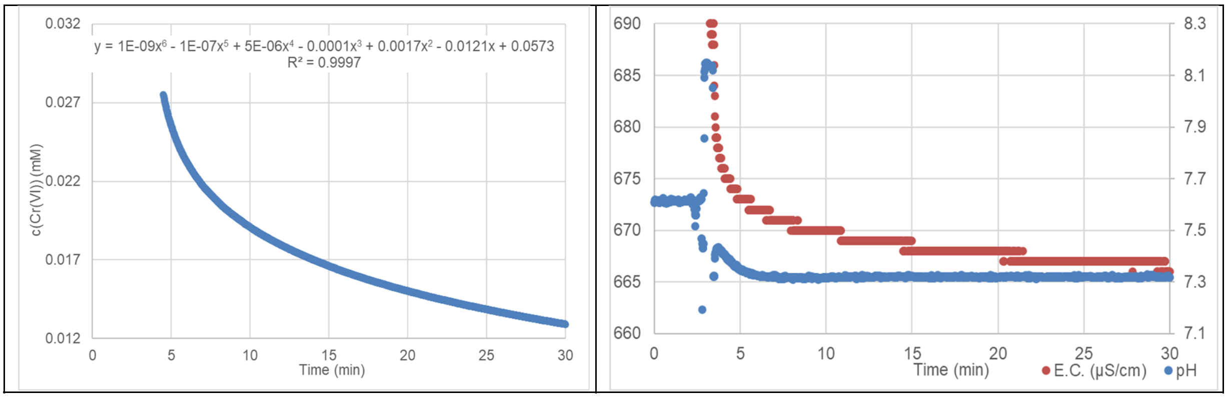

(a) (b)

Figure S16.7. 250 ml DW with 0.04 mM K_2CrO_4, 325 mg GNP. (a) Concentration of Cr(VI) as a function of time; (b)E.C. and pH as a function of time, E.C. (left axis), pH (right axis).

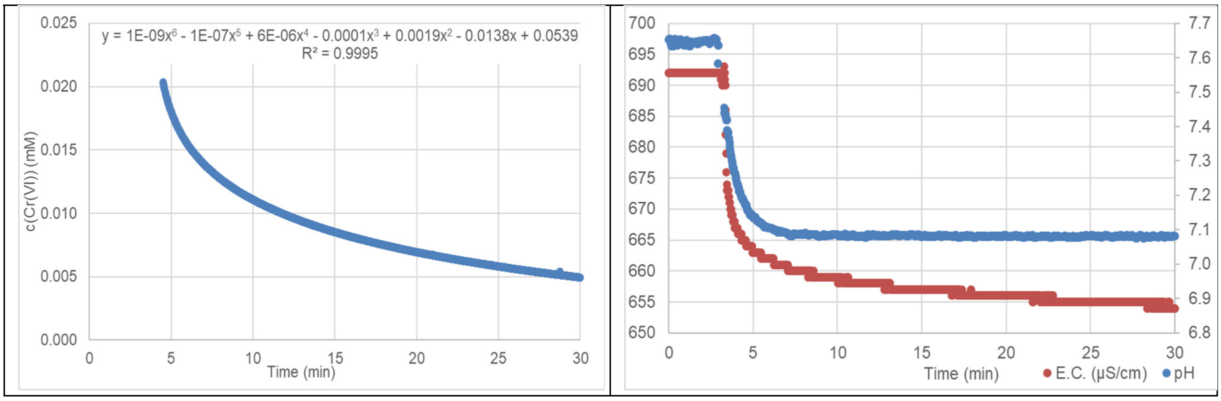

(a) (b)

Figure S16.8. 250 ml DW with 0.04 mM K_2CrO_4, 500 mg GNP. (a) Concentration of Cr(VI) as a function of time; (b)E.C. and pH as a function of time, E.C. (left axis), pH (right axis).

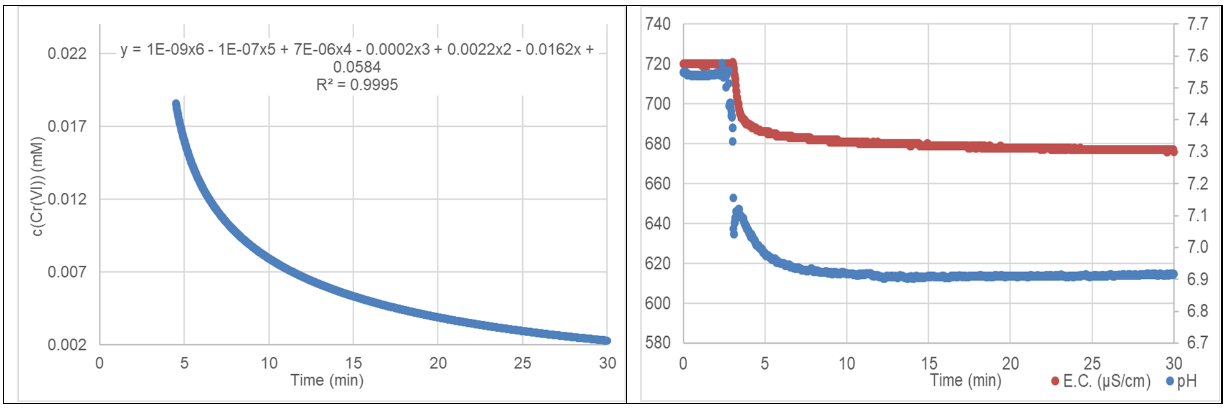

(a) (b)

Figure S16.9. 250 ml DW with 0.04 mM K_2CrO_4, 625 mg GNP. (a) Concentration of Cr(VI) as a function of time; (b)E.C. and pH as a function of time, E.C. (left axis), pH (right axis).

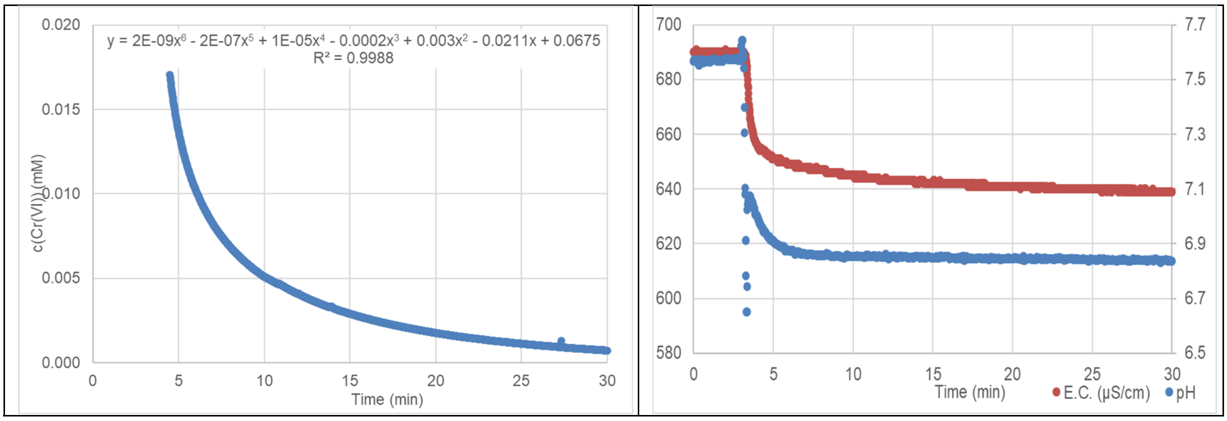

(**a**) (**b**)

Figure S16.10. 250 ml DW with 0.04 mM K_2CrO_4, 750 mg GNP. (a) Concentration of Cr(VI) as a function of time; (b)E.C. and pH as a function of time, E.C. (left axis), pH (right axis).

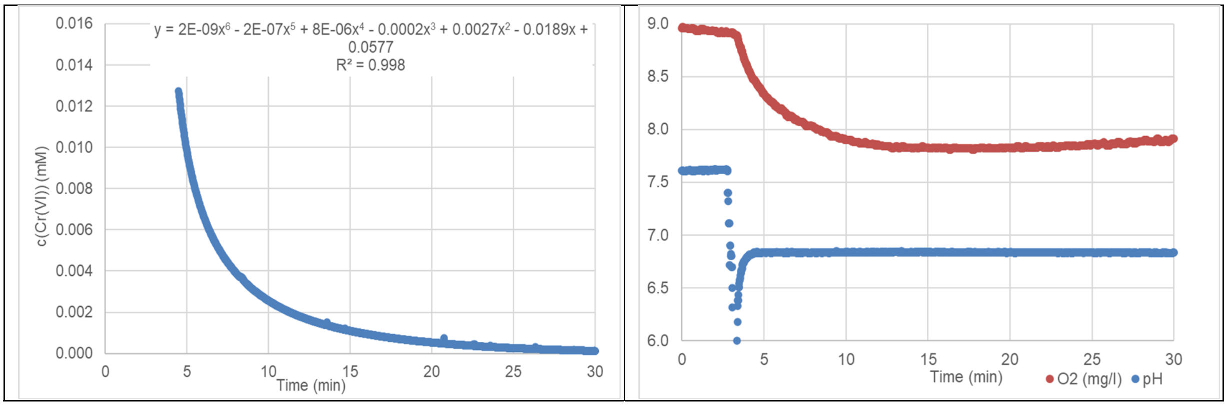

(**a**) (**b**)

Figure S16.11. 250 ml DW with 0.04 mM K_2CrO_4, 875 mg GNP. (a) Concentration of Cr(VI) as a function of time; (b)E.C. and pH as a function of time, E.C. (left axis), pH (right axis).

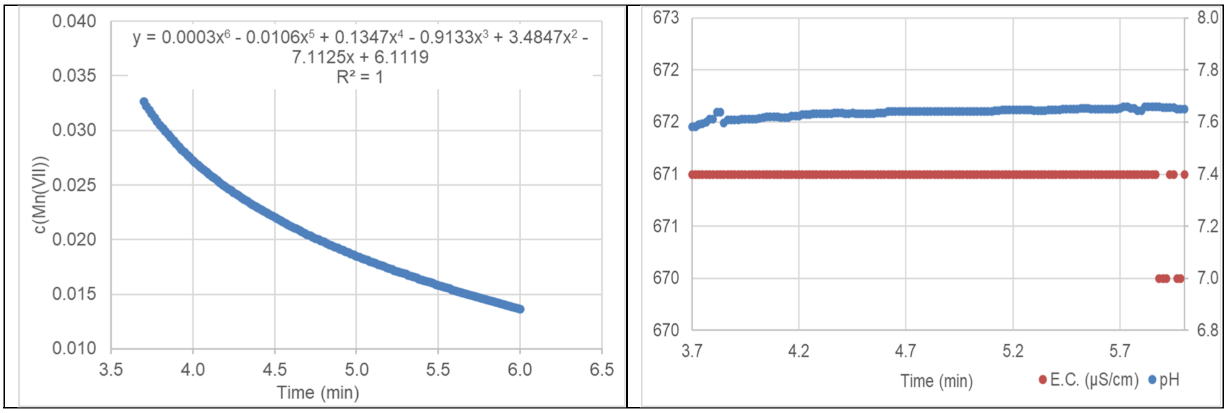

(**a**) (**b**)

Figure S16.12. 250 ml DW with 0.04 mM $KMnO_4$, 10 mg GNP. (a) Concentration of Mn(VII) as a function of time; (b)E.C. and pH as a function of time, E.C. (left axis), pH (right axis).

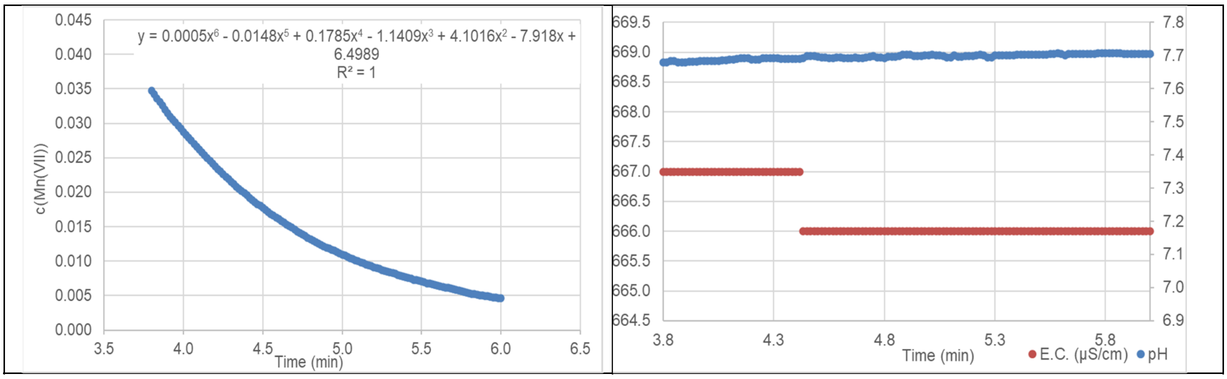

(**a**) (**b**)

Figure S16.13. 250 ml DW with 0.04 mM $KMnO_4$, 20 mg GNP. (a) Concentration of Mn(VII) as a function of time; (b)E.C. and pH as a function of time, E.C. (left axis), pH (right axis).

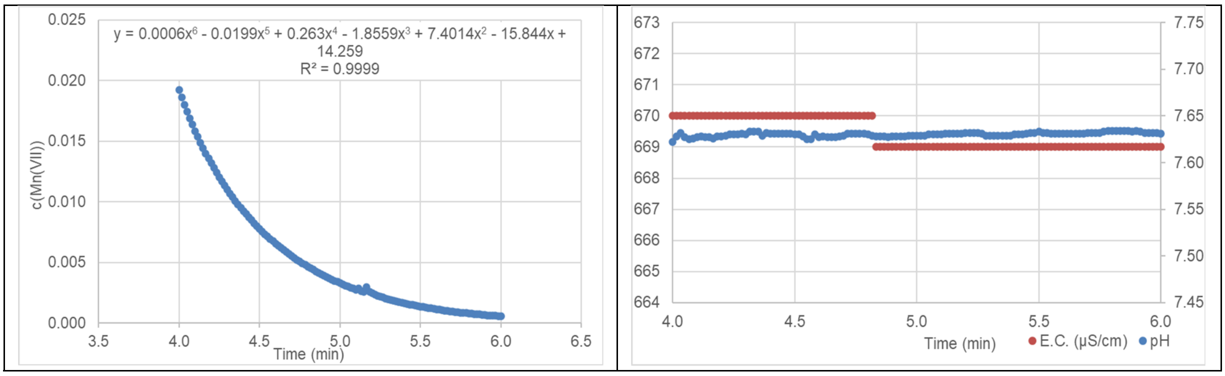

(**a**) (**b**)

Figure S16.14. 250 ml DW with 0.04 mM $KMnO_4$, 30 mg GNP. (a) Concentration of Mn(VII) as a function of time; (b)E.C. and pH as a function of time, E.C. (left axis), pH (right axis).

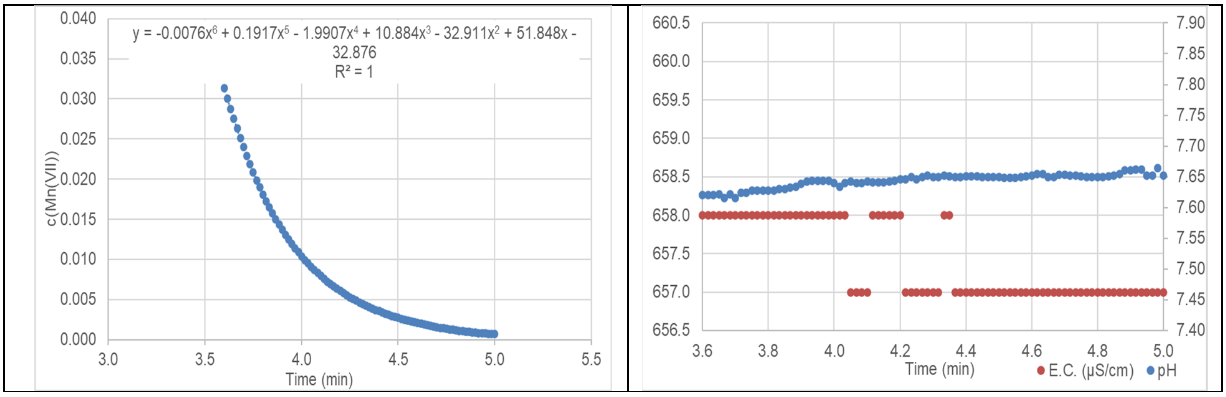

(**a**) (**b**)

Figure S16.15. 250 ml DW with 0.04 mM $KMnO_4$, 40 mg GNP. (a) Concentration of Mn(VII) as a function of time; (b)E.C. and pH as a function of time, E.C. (left axis), pH (right axis).

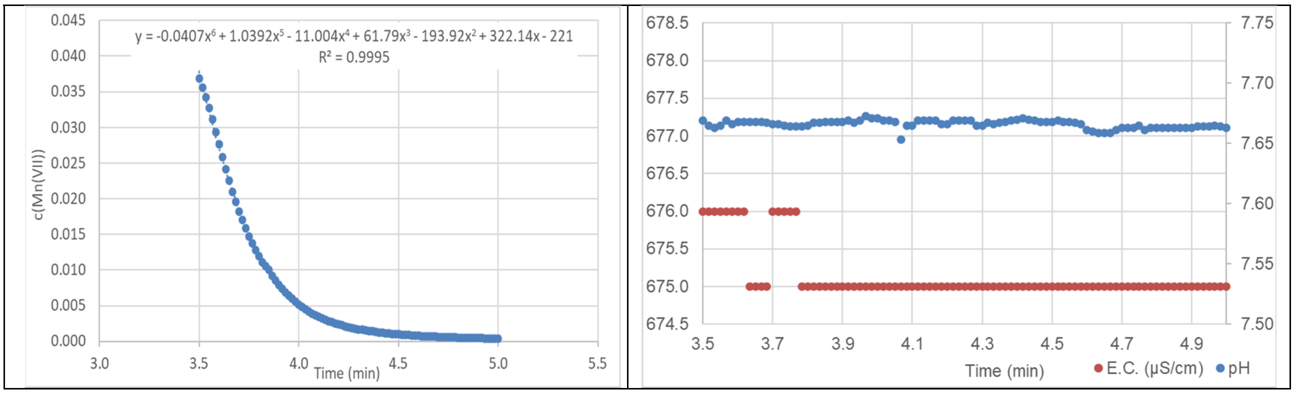

(**a**) (**b**)

Figure S16.16. 250 ml DW with 0.04 mM $KMnO_4$, 50 mg GNP. (a) Concentration of Mn(VII) as a function of time; (b)E.C. and pH as a function of time, E.C. (left axis), pH (right axis).

S17. Evaluation of the influence of GNP mass on oxometalate sorption kinetics.

Table S17.1. PSO adsorption kinetic evaluation of 0.04 mM vanadium(V) in DW with different GNP mass.[1]

Sorption	T_m (°C)	k_{PSO} ($mM^{-1} min^{-1}$)	q_e ($mM L^{-1}$), calculated	q_e ($mM L^{-1}$), exp.[1]	R^2 adj	pH start	ΔpH	ΔE.C. (μS/cm)
0.04 mM NH_4VO_3 in DW, 500 mg GNP	25.9 ± 0.2	30.5 ± 0.5	0.0169 ± 0.0001	0.00881 ± 0.0003	0.9857	6.96	-0.02	-4
0.04 mM NH_4VO_3 in DW, 625 mg GNP	24.8 ± 0.1	25.4 ± 0.4	0.0195 ± 0.0001	0.00448 ± 0.0001	0.9881	6.84	0.00	-4
0.04 mM NH_4VO_3 in DW, 750 mg GNP	22.9 ± 0.2	25.8 ± 0.6	0.0221 ± 0.0001	0.00430 ± 0.0001	0.9737	6.93	-0.09	-7
0.04 mM NH_4VO_3 in DW, 875 mg GNP	22.9 ± 0.2	20.8 ± 0.5	0.0253 ± 0.0001	0.00267 ± 0.0001	0.9725	6.93	-0.09	-7
0.04 mM NH_4VO_3 in DW, 1000 mg GNP	25.6 ± 0.1	20.9 ± 0.5	0.025 ± 0.0001	0.00207 ± 0.0001	0.9752	6.72	-0.07	-7

[1] Data for regression: 31, evaluation time: 7.0 – 18.0; [2] ICP/MS values at equilibrium.

Table S17.2. Pseudo-second-order kinetic evaluation of 0.04 mM chromium(VI) in DW with different GNP mass.

Sorption	Data for regression	T_m (°C)	Evaluation time (min)	k_2 (mM^{-1} *min^{-1})	R^2 adj	pH start	ΔpH	ΔE.C. (μS/cm)
0.04 mM K_2CrO_4 in DW, 250 mg GNP	19	24.2 ± 0.1	7.0 - 9.0	1.25 ± 0.10	0.9466	7.26	0.00	0
0.04 mM K_2CrO_4 in DW, 325 mg GNP	20	22.9 ± 0.1	6.9 - 9.4	2.20 ± 0.14	0.9616	7.32	0.00	-1
0.04 mM K_2CrO_4 in DW, 500 mg GNP	20	24.9± 0.1	6.7 - 9.0	6.35 ± 0.24	0.9867	7.09	0.00	-2
0.04 mM K_2CrO_4 in DW, 625 mg GNP	24	24.5 ± 0.1	6.7 - 9.0	12.1 ± 0.2	0.9976	6.94	-0.02	-2
0.04 mM K_2CrO_4 in DW, 750 mg GNP	21	24.8 ± 0.1	5.0 - 9.0	24.1 ± 0.3	0.9982	6.91	-0.05	-5
0.04 mM K_2CrO_4 in DW, 875 mg GNP	30	24.3 ± 0.1	4.0 - 7.0	48.2 ± 1.0	0.9905	6.80	0.04	-

Table S17.3. Pseudo-first-order kinetic evaluation of 0.04 mM manganese(VII) in DW with different GNP mass.

Process	Data for regression	T_m (°C)	Evaluation time (min)	k_1 (min^{-1})	R^2 adj	pH start	ΔpH	ΔE.C. (μS/cm)
0.04 mM $KMnO_4$ in DW, 10 mg GNP	27	25.1 ± 0.1	4.2 - 5.9	0.333 ± 0.022	0.9230	7.62	0.03	-1
0.04 mM $KMnO_4$ in DW, 20 mg GNP	25	25.7 ± 0.1	4.2 - 5.9	0.924 ± 0.031	0.9830	7.69	0.02	-1
0.04 mM $KMnO_4$ in DW, 30 mg GNP	25	25.1 ± 0.1	4.2 - 5.9	1.74 ± 0.02	0.9985	7.63	0.01	-1

0.04 mM $KMnO_4$ in DW, 40 mg GNP	21	25.0 ± 0.1	3.6 - 4.6	2.72 ± 0.03	0.9988	7.62	0.03	-1
0.04 mM $KMnO_4$ in DW, 50 mg GNP	19	25.3 ± 0.1	3.5 - 4.4	3.92 ± 0.02	0.9631	7.67	0.00	-1

Table S17.4. Error estimation for plotting log k_{PSO} of V(V) versus surface area of GNP. [1]

GNP (mg)	Area (m^2)	Pos. error area (m^2)	Neg. error area (m^2)	k_{PSO} (mM^{-1} *min^{-1})	Error k_{PSO} (mM^{-1} *min^{-1})	log k_{PSO}	Pos. error log k_2 (-)	Neg. error log k_2 (-)
500	375	14	11	30.5	0.5	1.4843	0.01	0.01
625	469	16	13	25.4	0.4	1.4048	0.01	0.01
750	563	19	15	25.8	0.6	1.4116	0.02	0.02
875	656	21	17	20.8	0.5	1.3181	0.02	0.02
1000	750	24	19	20.9	0.5	1.3201	0.02	0.02

[1] Error in weighting and entry of GNP of estimated 5 mg results in 3.8 m^2 error in surface area. Errors smaller than 1.0 mM^{-1} min^{-1} are rounded up to 1.0 mM^{-1} min^{-1}.

Table S17.5. Error estimation for plotting log k_2 of Cr(VI) sorption versus surface area of GNP. [1]

GNP (mg)	Area (m^2)	Pos. error area (m^2)	Neg. error area (m^2)	k_2 (mM^{-1} *min^{-1})	Error (mM^{-1} *min^{-1})	log k_2	Pos. error log k_2 (-)	Neg. error log k_2 (-)
250	188	9	9	1.25	0.10	0.0969	0.07	0.07
325	244	10	10	2.20	0.14	0.3424	0.06	0.05
500	375	14	14	6.35	0.24	0.8028	0.03	0.03
625	469	16	16	12.1	0.20	1.0828	0.01	0.01
750	563	19	19	24.1	0.3	1.3820	0.01	0.01
875	656	21	21	48.2	1.0	1.6830	0.02	0.02

[1] Error in weighting and entry of GNP of estimated 5 mg results in 3.8 m^2 error in surface area. Errors smaller than 1.0 mM^{-1} min^{-1} are rounded up to 1.0 mM^{-1} min^{-1}.

Table S17.6. Error estimation for plotting k_1 of Mn(VII) sorption versus surface area of GNP. [1]

GNP (mg)	Area (m^2)	Error area (m^2)	k_1 (min^{-1})	Error (mM^{-1} *min^{-1})	log (area)	Neg. error log area (-)	Pos. error log area (-)	log k_1	Neg. error log k_1 (-)	Pos. error log k_1 (-)
10	7.5	1.2	0.333	0.0792	0.875	0.076	0.064	-0.478	0.118	0.093
20	15.0	1.4	0.924	0.1461	1.176	0.043	0.039	-0.034	0.075	0.064
30	22.5	1.6	1.74	0.2041	1.352	0.032	0.030	0.241	0.054	0.048
40	30.0	1.8	2.72	0.2553	1.477	0.027	0.025	0.435	0.043	0.039
50	37.5	2.0	3.92	0.3010	1.574	0.024	0.023	0.593	0.035	0.032

[1] Error in weighting and entry of GNP of estimated 1 mg results in 0.8 m^2 error in surface area.

S18. Simultaneous sorption of V(V) and Cr(VI) on GNP.

Table S18. Simultaneous kinetics of 0.04 mM NH_4VO_3 and 0.04 mM K_2CrO_4 in DW, 510.9 mg GNP.

Time (min)	Abs. 263 nm	Abs. 263 nm corr.	Abs. 375 nm	$c(VO_3)$ corr. (mM)	$c(CrO_4)$ (mM)	$1/c(VO_3)$ (1/mM)	$1/c(CrO_4)$ (1/mM)	$q(t)$ VO_3 (mM min^{-1})	$1/q(t)$ VO_3 (min/mM)
0.0	1.002	0.330	0.922	0.040	0.0400	25.00	25.00	0.0000	
1.0	0.637	0.210	0.586	0.025	0.0254	39.33	39.31	0.0146	68.63
1.8	0.554	0.214	0.467	0.026	0.0203	38.64	49.30	0.0141	70.84
2.5	0.516	0.211	0.417	0.026	0.0181	39.04	55.21	0.0144	69.51
3.2	0.486	0.208	0.382	0.025	0.0166	39.73	60.35	0.0148	67.43
4.0	0.460	0.205	0.350	0.025	0.0152	40.30	65.92	0.0152	65.86
6.0	0.416	0.198	0.298	0.024	0.0129	41.58	77.34	0.0159	62.70
10.0	0.362	0.191	0.235	0.023	0.0102	43.24	97.93	0.0169	59.26
15.0	0.322	0.185	0.188	0.022	0.0082	44.66	122.4	0.0176	56.79
22.0	0.291	0.180	0.152	0.022	0.0066	45.88	151.4	0.0182	54.93
30.0	0.255	0.175	0.110	0.021	0.0048	47.13	209.0	0.0188	53.24

Spectra were recorded from 238 - 398 nm and the absorptions at 263 nm and 375 nm were evaluated.

Corrections were made for the V(V) absorption band using the fixed absorption band ratio of Cr(VI) at 263 and 375 nm of 0.7288. To do this, the time-dependent absorption band at 375 nm was multiplied by the factor and the corresponding value was subtracted from the band at 263 nm to obtain the absorption of V(V).

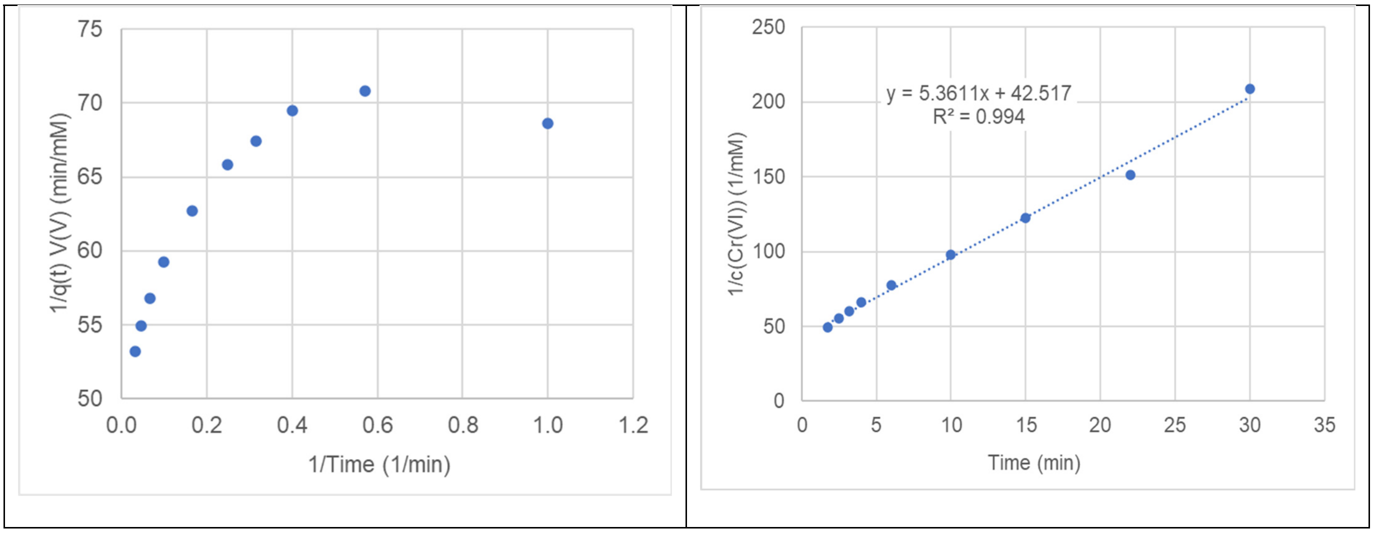

(a) (b)

Figure S18. Concurrent sorption of V(V) and Cr(VI). (a) PSO plot for V(V); (b) Pseudo-second-order plot for Cr(VI).

S19. XRD of the reaction product of GNP with manganese(VII).

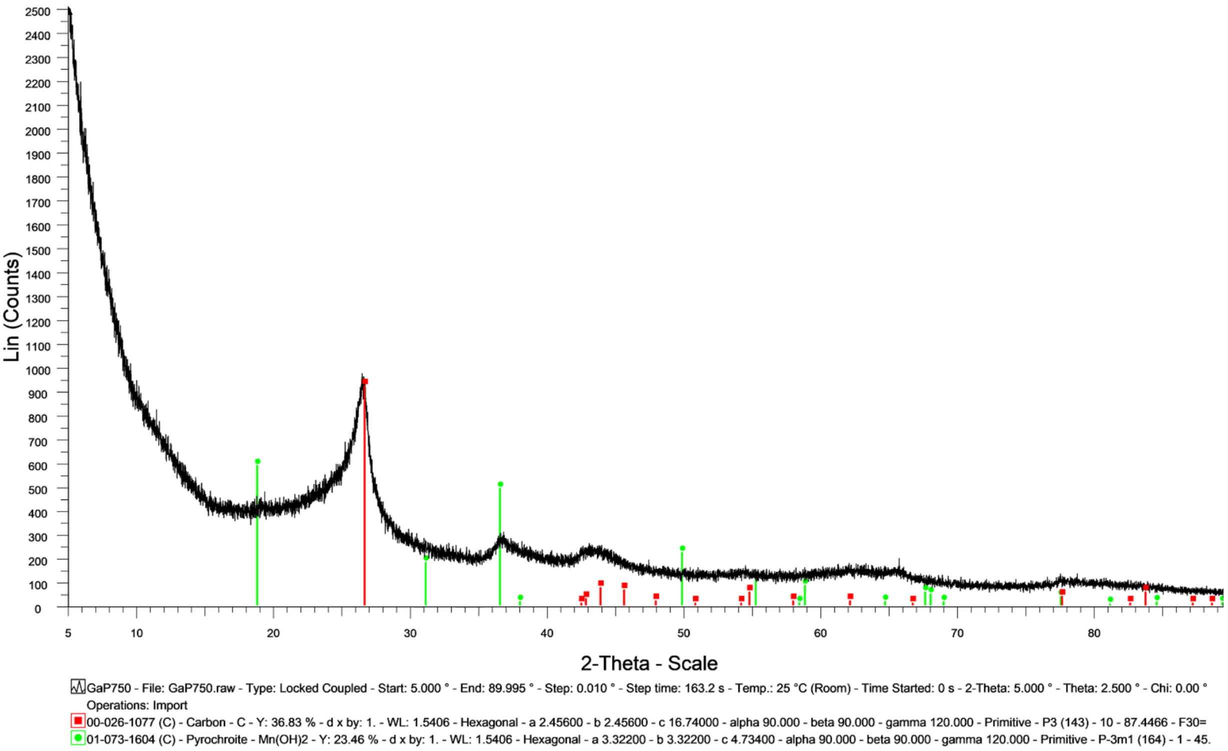

Figure S19. XRD of Mn-GNP.

S20. ORP measurements of 0.04 mM oxometalate in drinking water.

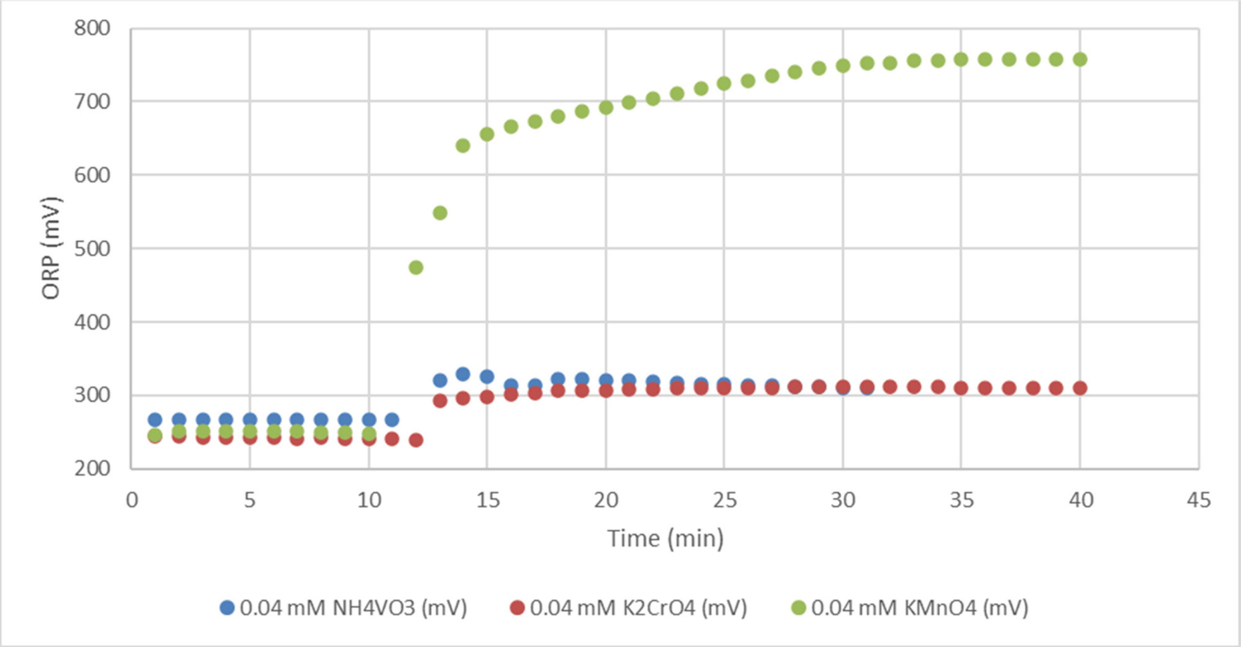

Figure S20. Time-dependent ORP measurements before and after dosage of 0.04 mM oxometalate in air-saturated DW with stirring (200 rpm), temperature 22.2 ± 1.2 °C.
